# Medicinal Mushrooms in Colon Cancer Therapy: Mechanisms of Action of Bioactive Compounds and Therapeutic Potential

**DOI:** 10.3390/ijms26115304

**Published:** 2025-05-31

**Authors:** Jinangi Bentharavithana, Tahidul Islam, Baojun Xu

**Affiliations:** 1 Food Science and Technology Program, Department of Life Sciences, Beijing Normal-Hong Kong Baptist University, Zhuhai 519085, China; jinangibv97@gmail.com (J.B.); nobab01@hkbu.edu.hk (T.I.); 2 Department of Food Science and Technology, Faculty of Agriculture, University of Peradeniya, Peradeniya 20400, Sri Lanka; 3 School of Chinese Medicine, Hong Kong Baptist University, Hong Kong SAR, China

**Keywords:** apoptosis, bioactive compounds, clinical study, edible mushrooms, medicinal mushrooms

## Abstract

Colon cancer is the second leading cause of cancer-related deaths in the world. This is commonly observed among older adults, and the occurrence of colon cancer is mainly influenced by unhealthy lifestyle factors. Edible medicinal mushrooms have been demonstrated to have anti-colon cancer effects both individually and in combination with conventional therapies, including synergistically enhancing the efficacy of chemotherapy medications such as 5-fluorouracil in preclinical models. Medicinal mushrooms such as *Lentinus edodes*, *Phellinus linteus*, *Ganoderma lucidum*, *Inonotus obliquus*, *Pleurotus ostreatus*, *Hericium erinaceus*, *Pleurotus eryngii*, *Gloeostereum incarnatum*, and *Termitomyces heimii* are emerging as promising candidates, not only because conventional treatments for colon cancer face significant limitations, including side effects, psychological impacts on patients, high cost, limited specificity toward cancer and healthy cells, and the development of drug resistance, but also due to the diverse array of bioactive compounds present within them. Therefore, there is a strong demand for innovative, affordable, and minimally invasive treatments such as medicinal mushrooms. Their bioactive compounds, including terpenoids, sterols, phenols, polysaccharides, acids, sesquiterpenes, alkaloids, lactones, metal-chelating agents, nucleotide analogs, glycoproteins, β-glucan, cerebrosides, steroids, terpenes, quinolones, anthraquinones, benzoic acid derivatives, linoleic acid, ascorbic acid, glycosides, organic acids, flavonoids, grifolin, tocopherols, proteins, indoles, lectin, and laccases, exert anti-colon cancer activities through various mechanisms, including anti-proliferative effects, cell cycle arrest, anti-inflammatory effects, antioxidant effects, induction of apoptosis, cytotoxic effects, and antimigratory effects. Further research is needed to elucidate the molecular mechanisms and confirm the safety and efficacy of medicinal mushrooms as a holistic anti-colon cancer treatment.

## 1. Introduction

Mushrooms are filamentous fungi with fruiting bodies. For a long time, they have been an essential component in the human diet as well as in medicine and pharmacology [1]. The Chinese and Japanese have utilized mushrooms for medicinal purposes for thousands of years. Nowadays, mushrooms are popular, valuable foods because they are low in calories, carbohydrates, fat, and sodium and are cholesterol-free. Besides, mushrooms provide important nutrients, including selenium, potassium, riboflavin, niacin, vitamin D, proteins, and fiber [2]. There are many health-promoting functionalities found in mushrooms and it has been reported that more than 100 medicinal functions are produced by mushrooms and fungi, which have antioxidant, anticancer, antidiabetic, antiallergic, immunomodulating, cardiovascular protector, anticholesterolemic, antiviral, antibacterial, antiparasitic, antifungal, detoxification, and hepatoprotective effects, and also protect against tumor development and inflammatory processes [3,4,5,6]. Colorectal cancer is the third most commonly diagnosed cancer and the fourth most common cause of cancer-related mortality globally. The highest incidence and mortality rates are seen in high-income countries due to unhealthy diets [7]. However, other factors such as a history of polyps in the colon, age over 50 years, a personal history of inflammatory bowel disease, family colon cancer history, obesity, low exercise, red meat consumption, smoking, and alcohol consumption are driving factors for the growth of colon cancer. The incidence of colorectal cancer is especially increased in developing countries. It has been reported that nearly 2 million new cases and about 1 million deaths are expected in 2018 [8], which demonstrates that only 50% of colon cancer patients could survive. Treatments such as chemotherapy, surgery, radiation therapy, targeted therapy, and immunotherapy may damage healthy cells and tissues and cause side effects in the body. Therefore, researchers are exploring natural alternative methods with minimum side effects. This review focuses on medicinal mushrooms as an alternative and their different molecular mechanisms in lab, animal, and human studies against colon cancer.

## 2. Edible and Medicinal Mushrooms with Anti-Colon Cancer Effects

### 2.1. Definition of Medicinal and Edible Mushrooms

In many in vivo, in vitro, and clinical studies, medicinal mushroom consumption has shown an inverse correlation with gastrointestinal cancer occurrence [9]. According to the Oxford Dictionary, the term ‘medicinal’ means having healing, curative, or therapeutic attributes or used as a medicine or related to medicine. The term ‘edible’ means fit to be eaten. Medicinal mushrooms are macroscopic fungi belonging to the phylum Basidiomycota. These can be consumed as food items and used as extracts or powders to treat diseases. Bioactive compounds present in medicinal mushrooms enhance their medicinal and nutraceutical properties. Medicinal mushrooms are rich in a plethora of biologically active secondary metabolites that possess beneficial health effects such as anticancer, antimicrobial, antiviral, anti-inflammatory, antidiabetic, and antioxidant effects. Even though there are 140,000 mushroom species available in nature, only 2000 species are in edible form. Among the edible forms, only 25 species are commercially available [10,11]. There are around 2000 mushrooms that show medicinal properties, and over 600 have been confirmed with official data [12]. Edible mushrooms show medicinal properties through anticancer, antiviral, hepatoprotective, anti-cardiovascular disease, immunopotentive, antioxidative, and hypocholesterolemic effects. Therefore, many research techniques are involved in extracting the bioactive compounds present in edible mushrooms that show medicinal properties [13]. Edible mushrooms are used to prepare drugs and nutraceuticals that have antitumor, antioxidant, and antimicrobial effects. The low starch, low fat, and high fiber contents of edible mushrooms make them ideal food items for both obese and diabetic patients. Due to this, edible mushrooms are emerging as potent sources of pharmaceuticals [14]. *Letinula edodes*, *Ganoderma Lucidum*, *Grifola frondosa*, *Trametes versicolor*, and *Inonotus obliquus* are popular edible medicinal fungi that have been used in both traditional and modern medicine and research [12]. There are both edible medicinal mushroom species and inedible medicinal mushroom species. *Heterobasidion annosum* and *Trametes versicolor* are medicinal mushrooms that can be used as potential medicines for colorectal cancer treatment, but they are not edible [15,16].

### 2.2. Types of Edible and Medicinal Mushrooms with Anti-Colon Cancer Properties

**Figure 1:**
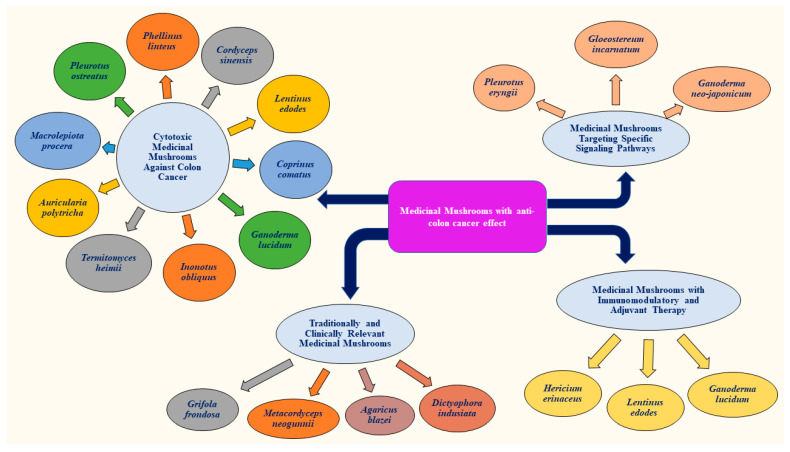
Edible and medicinal mushrooms with anti-colon cancer effects.

Mushrooms are becoming more interesting due to their dual edible and medicinal nature [17]. The bioactive compounds present in medicinal mushrooms demonstrate anticancer activity through various mechanisms. This section classifies key edible and medicinal mushrooms (MMs) according to their demonstrated anti-colon cancer effects and usage. Figure 1 presents edible and medicinal mushrooms with anti-colon cancer effects.

#### 2.2.1. Potent Cytotoxic Medicinal Mushrooms Against Colon Cancer

Meshima (*Phellinus linteus* (Berk. et Curt.) Teng), caterpillar fungus (*Cordyceps sinensis* (Berk.) Sacc.), shiitake mushrooms (*Lentinus edodes* (Berk.) Pegler), shaggy ink cap (*Coprinus comatus* (O.F. Müll.) Pers.), and lingzhi/reishi (*Ganoderma lucidum* (Curtis) P. Karst) are some of the traditional and commercially available edible MMs with anti-colon cancer properties. Methanolic extracts of *Phellinus linteus* dramatically decreased the cell viability of human colorectal carcinoma cell line 116 (HCT-116) and surg pathol-derived colorectal cancer cell line 480 (SW-480). Other mushroom varieties showed selective inhibition of SW-480 cancer cell line viability. The MTT assay showed a broad spectrum of cytotoxic effects induced by *Phellinus linteus* when compared to the other four varieties [18]. Research conducted using a hot water extract of *Inonotus obliquus* (IOWE) showed that it can inhibit the growth of human colorectal adenocarcinoma cell line 29 (HT-29) in a dose-dependent manner. The most effective IOWE concentration was 1.0 mg/mL for 48 h, with a maximum inhibitory activity of 56% [6]. *Termitomyces heimii* showed an in vitro cytotoxic effect against the HCT-116 colon cancer cell line [9]. *Auricularia polytricha*, *Macrolepiota procera*, and *Pleurotus ostreatus* are edible and medicinal mushrooms with anti-colon cancer effects [19]. A study conducted on the anticancer properties of *Pleurotus ostreatus* grown in selenium (Se) showed anti-colon cancer effects against a human colorectal adenocarcinoma cell line (Caco-2) and normal human colon mucosal epithelial cancer cell line (NCM-460). These findings provide insight into the anti-colon cancer effects of selenium-rich fruiting bodies of edible medicinal mushrooms [20].

#### 2.2.2. Medicinal Mushrooms with Immunomodulatory Effects and Adjuvant Therapy

*Lentinus edodes* is one of the most popular edible mushrooms used as a food in Eastern countries. The *Lentinus edodes* extract has shown its potential as a novel chemotherapeutic adjuvant agent when combined with 5-fluorouracil [21]. *β*-Glucan from *Lentinus edodes* showed promising results for colitis-associated colorectal cancer and the gut microbiota [22]. *Ganoderma lucidum* is another edible MM that has an inhibitory effect against colorectal cancer. In vitro co-administration of a non-toxic concentration (0.3 mg/mL) of an extract of a commercial product that contained spores and fruiting bodies (30:8 ratio) of *Ganoderma lucidum* (GLSF) and paclitaxel, a chemotherapy medication (0.125 μM), showed significant cancer cell growth inhibition and apoptosis in the CT26 murine colon carcinoma cell line and HCT-15 human colon cancer cell line. In vivo studies showed that CT26 tumor cell growth was suppressed when mice were orally administered a modified diet that contained 1.25% GLSF powder [23]. *G. lucidum* supplements are used by cancer patients to boost their immune system. This mushroom’s fruiting bodies, mycelia, or spores contain anticancer chemical compounds, particularly polysaccharides and triterpenes [24]. Since ancient times in China, *Hericium erinaceus*, also known as lion’s mane, has been used to treat digestive system disorder-related diseases. This medicinal mushroom studied worldwide has anticancer effects against colon cancer cells [25].

#### 2.2.3. Medicinal Mushrooms Targeting Specific Signaling Pathways

*Gloeostereum incarnatum* (GI) is an edible mushroom with anti-colon cancer activity. Its water-soluble polysaccharide extract (GIPS) shows anti-colon cancer activity through the Wnt signaling pathway [26]. *Ganoderma neo-japonicum* Imazeki was identified as a potential natural anticancer agent against human colon cancer through cellular and computational models [27]. *Pleurotus eryngii,* also known as king trumpet mushroom or French horn mushroom, is an edible mushroom that showed inhibitory activity against the growth of HCT-116 colon cancer cells and an anti-inflammatory function in the RAW264.7 macrophage cell line [28].

#### 2.2.4. Traditionally and Clinically Relevant Medicinal Mushrooms

*Inonotus obliquus* (Chaga) is an edible medicinal mushroom with antioxidant, anti-inflammatory, antitumor, immunomodulatory, and antimutagenic activities. The toxicity associated with improper usage of Chaga is now addressed by toxicity assessments, risk analyses, and guidelines for proper usage [29]. In vitro research conducted on n-hexane extracts of *Hericium erinaceus*, *Metacordyceps neogunnii*, and *Dictyophora indusiata* showed that these edible and medicinal mushrooms exerted anti-colon cancer effects against the HCT-116 human colon cancer cell line [30]. The raw form of *Ganoderma lucidum* medicinal mushroom is not edible, but powders, dietary supplements, and tea are commercially available products of this mushroom. This species is renowned for its pharmaceutical activities rather than its nutritional value. A water extract of sporoderm-broken spores of *Ganoderma lucidum* showed an anticarcinogenic effect against colorectal cancer [31]. *Lentinus edodes* shows its anticancer activity by activating immune responses, prohibiting colon cancer cell proliferation, suppressing tumor growth, and inducing cell apoptosis [32]. It also demonstrates antioxidant, antidiabetic, antihepatitic, hyperlipidemic, antifungal, immunomodulatory, antibacterial, antiviral, and antitumor activities [33]. The water extracts of the mycelium of *Agaricus blazei* (82.4%), *Hericeum erinaceus* (14.7%) and *Grifola frondosa* (2.9%), collectively called Andosan™, a medicinal mushroom extract, showed their potential as a natural preventive and therapeutic agent for colorectal cancer in an A/J Min/+ mice model [34,35].

### 2.3. Chemical Compositions of Mushrooms with Anti-Colon Cancer Properties

Edible and medicinal mushrooms are generally low in calories, lipids, and cholesterol and have high contents of protein and essential vitamins. The fruiting bodies of edible and medicinal mushrooms contain a variety of secondary [11,14]. Edible mushrooms are usually low in fat and energy while high in protein. Generally, many edible mushrooms that show medicinal properties are rich in minerals (iron, phosphorus) and vitamins (riboflavin, thiamine, ergosterol, niacin, ascorbic acid). *β*-Glucans and glycoproteins are the main polysaccharides present in edible and medicinal mushrooms. Terpenoids, acids, alkaloids, sesquiterpenes, lactones, sterols, metal-chelating agents, vitamins, nucleotide analogs, and polyphenolic compounds are the main secondary metabolites present in these mushrooms [13]. Chitin and starch are the main carbohydrates present in edible and medicinal mushrooms. Many medicinal mushrooms are good sources of crude fiber such as chitin, mannan, and hemicellulose. The nutrient, enzyme, bioactive compound, vitamin, and mineral contents vary with the type of edible medicinal mushroom [12]. Edible and medicinal mushrooms are rich sources of selenium, potassium, riboflavin, niacin, vitamin D, proteins, and fiber. Also, they are free from cholesterol and contain low calories and fat, sodium, and carbohydrate contents. Therefore, these edible and medicinal mushrooms are used to treat different diseases, including cancer, and to improve human health [2].

### 2.4. Bioactive Compounds in Edible and Medicinal Mushrooms with Anti-Colon Cancer Effects

Edible and medicinal mushrooms are good sources of biologically active compounds such as terpenoids, sterols, phenols, and polysaccharides [2,12,28]. The bioactive molecules can be categorized as high-molecular-weight compounds (polysaccharides and proteins) and low-molecular-weight compounds (indoles, terpenoids, and phenols) [9]. There are secondary metabolites, glycoproteins, and polysaccharides. The secondary metabolites include acids, terpenoids, polyphenols, sesquiterpenes, alkaloids, lactones, sterols, metal-chelating agents, nucleotide analogs, and vitamins. *β*-Glucan is the main polysaccharide present in edible MMs [2]. Several bioactive polysaccharides and polysaccharide–protein complexes also play significant roles in cancer treatment [36]. The bioactive compounds in *Hericium erinaceus*, such as terpenoids, polyphenols, cerebrosides, and polysaccharides, have shown a variety of potential therapeutic applications such as antioxidant, immune-modulatory, and anticancer properties [25]. The secondary metabolites in medicinal mushrooms, such as steroids, terpenes, quinolones, anthraquinones, and benzoic acid derivatives, have shown anti-carcinogenesis effects. In addition, linoleic acid, ascorbic acid, polysaccharides, glycosides, organic acids, flavonoids, phenols, grifolin, and tocopherols exert their metabolic activities by actively scavenging nitrite radicals, hydroxyl radicals, and oxygen free radicals, which aids in suppressing cancer cell proliferation. Bioactive compounds in medicinal mushrooms demonstrate their anticancer activities through multiple mechanisms, including inhibition of nuclear factor kappa-light-chain-enhancer of activated B cells (NF-κB) activation, inhibition of protein kinase B (Akt) processes, and inhibition of cyclooxygenase-2 (COX-2) expression [37].

#### 2.4.1. Polysaccharides as Bioactive Compounds in Edible and Medicinal Mushrooms

##### Anti-Colon Cancer Mechanisms of Medicinal Mushroom Polysaccharides

Polysaccharides are increasingly being recognized as the main active and multifunctional bioactive compounds in medicinal mushrooms. Medicinal mushroom-derived polysaccharides are being recognized as potential anticancer agents mainly due to their role in modulating the immune system [9,17,25]. In Chinese traditional medicine, *Ganoderma lucidum* polysaccharides (GLPs) are used for cancer prevention. Sporoderm-broken spores of *G. lucidum* water extract (BSGLWE) mainly contained GLPs [31]. A study conducted on *H. erinaceus* fruiting bodies extracted and purified a novel polysaccharide called HEFP-2b. HEFP-2b showed anti-colon cancer properties in HCT-116 and DLD1 colorectal adenocarcinoma cell line [25]. Lentinan extracted from *Lentinus edodes* is a clinically used *β*-1, 3-glucan polysaccharide with immunomodulatory and antitumor effects. A water-extracted polysaccharide from *Lentinus edodes* (SLNT) showed an anti-colon cancer effect in athymic nude mice by inhibiting human colon cancer cell proliferation and suppressing tumor growth [33]. Polysaccharides in *Agaricus bisporus*, *Antrodia camphorate*, *Boletus edulis*, *Cordyceps Sinensis*, *Flammulina velutipes, Lentinus edodes*, *Lignosus rhinoceros*, *Morchella esculenta*, and *Pleurotus eryngii* are active against colorectal carcinogenesis [37,38,39]. The polysaccharides extracted from *Agaricus bisporus* were encapsulated within alginate-kappa carrageenan microcapsules, and these microcapsules activated natural killer cells (NK cells), acting as a potential treatment for colon cancer [40]. *Agaricus blazei* Murill polysaccharide significantly enhanced the activity of CD8+ T cells, which aid in the killing of colorectal cancer cells, and effectively inhibited intraperitoneal tumors in mice. Therefore, *Agaricus blazei* Murill polysaccharide can be considered a promising therapeutic agent to treat colon cancer, particularly involving intraperitoneal dissemination [41].

##### Structural Diversity and Bioactivity of Polysaccharides in Medicinal Mushrooms

A homogeneous neutral polysaccharide, WAAP-1 (molecular weight of 10.1 kDa), extracted from *Agaricus bisporus* significantly inhibited HT-29 colon cancer cell proliferation. The monosaccharides present in WAAP-1 are glucose, mannose, and galactose in a molar ratio of 84.95:8.97:4.50, while the main chain is mainly composed of (1,4)-α-D-Glcp and (1,6)-*β*-D-Manp [17]. Polysaccharides are emerging as potential components to prevent and treat colorectal cancers. The very first research that found the *Agaricus bisporus* polysaccharide WAAP-2, with a triple-helical structure, tangled chain conformation, and a molecular weight of 121 kDa, demonstrated an anticancer colon effect against HT-29 cells. The primary structure of WAAP-2 contains mannose, glucose, and galactose at a molar ratio of 8.81:81.80:6.89 [42]. *Gloeostereum incarnatum* polysaccharide extract has shown anti-colon cancer effects in mice [43]. Anticancer research conducted using *Lentinus edodes* showed that its *β*-glucan fraction (lentinan) reduced inflammation, inhibited colon cancer cell proliferation, and induced colon cancer cell apoptosis. Among the three *β*-glucan fractions, the fraction with the lowest molecular weight showed the most promising anti-colon cancer activity by rebuilding the intestinal mucosal barrier [22].

α-Glucan, a low-molecular-weight carbohydrate, extracted from the hot water-soluble fraction of *Pleurotus ostreatus* mushroom showed anti-proliferative and pro-apoptotic effects against HT-29 colon cancer cells [44]. *Boletus edulis*, a medicinal mushroom having anti-colon cancer properties, contains carbohydrates as its major component, constituting 58.1% of the mushroom’s dry weight. The extract obtained using ohmic heating demonstrated a total sugar concentration of 437.5 mg/g extract, trehalose 341 mg/g, and rhamnose, mannitol, glucose, and fructose [45,46]. Lentinan, a polysaccharide with *β*-glucans, demonstrated immunomodulating properties and antitumor activity against human colorectal cancer [37,47,48].

Polysaccharide-K, which is also known as Krestin or PSK, is usually found in *Trametes versicolor* and shows anti-colon cancer properties. PSK is a complex molecule containing polysaccharides and proteins. The polysaccharides primarily contain *β*-glucans. The amino acid profile of PSK is rich in acidic amino acids, mainly glutamic and aspartic acids [37,49,50,51]. Maitake D-fraction is a complex compound derived from the Maitake mushroom (*Grifola frondosa*) that shows anti-colon cancer properties. This compound also contains *β*-glucans as the primary polysaccharide and forms a proteoglycan complex by binding proteins into the polysaccharide [37,52,53,54,55]. Schizophyllan, derived from *Schizophyllum commune*, is another promising polysaccharide with a *β*-glucan structure that shows anti-colon cancer properties [37,56,57].

##### Future Directions, Limitations, and Suggestions Concerning the Use of Polysaccharides in Medicinal Mushrooms

The anti-colon cancer effects of polysaccharides are exerted through diverse mechanisms, demonstrating the wide potential of mushroom polysaccharides. The structural diversity of polysaccharides allows them to act through multiple mechanisms rather than relying on a single pathway. This aligns well with modern colon cancer treatment strategies, as combination approaches improve therapeutic efficacy. Monosaccharide composition, linkage type, and molecular weight influence the structure–function relationship in anti-colon cancer activity. As a future direction, modifying polysaccharide branching patterns, molecular weight, and glycosidic linkages could enhance bioavailability. Developing nanoformulations may also improve oral bioavailability and tumor targeting. Despite being multifunctional bioactive agents, polysaccharides face challenges such as structural heterogeneity, poor bioavailability, and limited clinical research in humans. Additionally, variations in extraction methods can lead to inconsistent polysaccharide compositions.

#### 2.4.2. Proteins as Bioactive Compounds in Edible and Medicinal Mushrooms

A novel bioactive protein in *Pleurotus eryngii*, called ‘PEP’, exhibited anti-inflammatory and anti-colon cancer effects [58,59]. Protein extracts from *Calvatia lilacina*, *Pleurotus ostreatus*, and *Volvariella volvacea* showed anticancer effects against the SW480 and Tohoku Hospital Pediatrics-1 human monocytic leukemia (THP-1) cell lines. Each protein extract had a unique protein profile with a distinct molecular weight [60]. Low-carbohydrate proteins and peptides derived from mushrooms exhibit anticancer properties through unique mechanisms [61]. A protein fraction known as Cibacron blue affinity eluted protein (CBAEP) was isolated from *Termitomyces clypeatus*, *Pleurotus florida*, *Calocybe indica*, *Astraeus hygrometricus*, and *Volvariella volvacea*. CBAEP showed anti-proliferative activity in tumor cells, enhanced natural killer cell activity, and stimulated the proliferation of immune cells, particularly splenocytes, thymocytes, and bone marrow cells [62].

Lectin, a bioactive protein in *Agaricus bisporus* demonstrates anti-colon cancer activity by inhibiting cancer cell growth and stimulating the immune system [63,64,65,66]. Lectins in *Agrocybe aegerita* and *Grifola frondose* show anticancer activity by inducing apoptosis in cancer cells and stimulating the immune system, respectively [67]. Musarin, a polysaccharide peptide isolated from *Trametes versicolor* powder, downregulated the epidermal growth factor receptor-ras signaling pathway (EGFR-Ras signaling pathway) in colorectal cancer stem-like cells and inhibited the proliferation of CSC-like CD24+CD44+ HT29 cells [15,43]. Laccases are bioactive proteins that have shown promising anti-colon cancer activity through anti-proliferative and cytotoxic effects [5,66,68,69,70].

#### 2.4.3. Phenolic Compounds as Bioactive Compounds in Edible and Medicinal Mushrooms

##### Unique Attributes of Phenolic Compounds in Medicinal Mushrooms

Phenolic compounds present in certain mushroom species exhibit a wide range of biological activities. These findings have significant implications for the development of novel and effective mushroom-based anticancer therapies. Phenolic compounds are abundant in some mushrooms, which help exert diverse biological activities, including anti-colon cancer effects, and have antimicrobial, antioxidant, and anti-inflammatory properties [28,71,72]. In point of fact, phenolic compounds are present in many plants as well as mushrooms. Compared to plants, fungal phenolics are unique fungal-specific metabolites. Even though fungi are underutilized compared to plants, they have superior antioxidant activity. Fungi contain a diverse range of phenolic compounds that contribute to their antioxidant properties. Among species, the phenolic profile significantly varies, indicating species-specific uniqueness in biochemical composition. In addition, many fungi exhibit comparative or may even exhibit superior antioxidant activity in free radical scavenging. The extraction methods used to extract fungi influence the antioxidant activity of fungi. Therefore, fungi could offer unique phenolics and potentially higher bioactivity [73].

Fungi do not rely specifically on shikimate and phenylpropanoid pathways for the biosynthesis of phenolic compounds and they may have modified or alternative pathways. Most importantly, fungi modify their phenolic compounds using extracellular enzymes, which allow them to synthesize new hybrid molecules. Fungi molecules often demonstrate higher bioavailability in human cells [74]. A combination of different mushroom extracts exhibited superior cytotoxic effects against human colon cancer cell lines compared to single-species extracts due to the synergistic effects of both polyphenols and polysaccharides [75]. The study demonstrated the effectiveness of the combination of mushroom extracts against colon cancer cells compared to single-species extracts. As discussed above, fungi show species-specific uniqueness in biochemical composition, and interactions among these compounds may enhance the anti-colon cancer activity.

##### Anti-Colon Cancer Potential of Medicinal Mushroom-Derived Phenolics

A study was conducted on the phenolic compound concentrations and antioxidant activities of five edible and five medicinal mushrooms that are commonly cultivated in Korea to determine the DPPH radical scavenging activities. *Pleurotus eryngii* showed 15% activity when the reaction time was one minute and *Agaricus bisporus* showed 78% activity when the reaction time was 30 min. There were 28 phenolic compounds present in these 10 mushroom species. The average total concentrations of phenolic compounds in edible and medicinal mushrooms were 174 µg/g and 477 µg/g, respectively. The average total concentrations of flavonoids in edible and medicinal mushrooms were 22 µg/g and 76 µg/g, respectively [71]. Another study conducted on determining the total phenolic and flavonoid contents in *Agaricus bisporus*, *Boletus edulis*, *Calocybe gambosa*, *Cantharellus cibarius*, *Craterellus cornucopioides*, *Hygrophorus marzuolus*, *Lactarius deliciosus*, and *Pleurotus ostreatus* demonstrated that the total flavonoid concentrations and total phenolic concentrations ranged between 0.9 and 3 mg per gram dry matter (DM) and 1 and 6 mg per gram (DM), respectively. All 8 varieties of mushrooms contained homogentisic acid, free phenolic acid, and flavonoids such as myricetin and catechin [76].

*Termitomyces* mushroom species contain gallic acid, chlorogenic acid, caffeic acid, ellagic acid, catechins, epicatechins, rutin, isoquercitrin, quercitrin, quercetin, and kaempferol as phenolic compounds [72]. A polyphenol-rich extract was isolated from *Pleurotus eryngii* to study the anti-colon cancer activity and anti-inflammatory activity. The polyphenol-rich extract contained gallic acid monohydrate, 3-(3,4-dihydroxyphenyl)-propionic acid, methyl gallate, syringic acid, ellagic acid, and catechin. The polyphenol-rich extract exhibited both anti-inflammatory effects and inhibitory effects against human colon cancer cells [28]. The butanol fraction of *Ganoderma neo-japonicum* Imazeki showed the highest antioxidant activity against colonic carcinoma cells and the highest concentration of phenolic compounds [27]. A study demonstrated that the ethyl acetate fraction of *Pleurotus tuber-regium* mushroom sclerotium was rich in phenolic compounds, particularly chlorogenic acid and syringic acid. The ethyl acetate fraction demonstrated strong antioxidant activity by effectively scavenging free radicals in DPPH, ABTS, and hydrogen peroxide assays. In addition, it showed both in vivo and in vitro anti-angiogenic effects, suggesting a connection between the antioxidant properties and anti-angiogenic effects [77]. *Boletus edulis* is a medicinal mushroom with anti-colon cancer properties and its extract contains ellagic acid, rutin, and taxifolin at 532 µg/g, 465 µg/g, and 259 µg/g, respectively [46].

In general, medicinal mushrooms are rich in species-specific phenolic compounds and flavonoids that contribute to their anticancer activities. Medicinal mushrooms contain higher concentrations of these compounds than other non-medicinal mushrooms.

#### 2.4.4. Other Bioactive Compounds in Edible and Medicinal Mushrooms

Apart from major bioactive compounds, *Termitomyces* species have cerebrosides, ergostanes, fatty acid amides, serine, saponins, and proteases [72]. Both the hexane and chloroform fractions of *Ganoderma neo-japonicum* showed anti-colon cancer effects. These fractions were enriched with sterols and terpenoids, respectively. 1,25-Dihydroxyvitamin D3 3-glycoside, an active metabolite of vitamin D3, and stellasterol demonstrated strong binding affinities to B-cell lymphoma 2 (Bcl-2) anti-apoptotic protein and increased cell death in cancer cells [27]. The main active compound of the hexane extract of *Pleurotus sajor-caju* was the sterol ergosta-5,7,22-trien-3*β*-ol. In silico docking confirmed the anti-colon cancer effect by demonstrating the credible interaction between the active compound and Bcl-2 protein, a key protein that prevents apoptosis [4]. 4-Acetyl-antroquinonol B extracted from *Antrodia camphorata* inhibits colorectal cancer by suppressing tumorigenesis and cancer stem-like phenotype [78]. 4-Acetyl-antroquinonol B in mushrooms enhances the response of colorectal cancer cells to the drug cetuximab by inhibiting the KRAS signaling pathway [79,80]. Alkaloids, folate, enzymes, ergosterol, organic acids, and selenium are also bioactive compounds in mushrooms. Antroquinonol, polysaccharide, cordycepin, hispolon, lectins, krestin, sulfated polysaccharides, lentinan, and Maitake D Fraction are some of the anticancer components present in medicinal mushrooms [78,81].

## 3. Mechanisms of Action Against Colon Cancer

**Figure 2:**
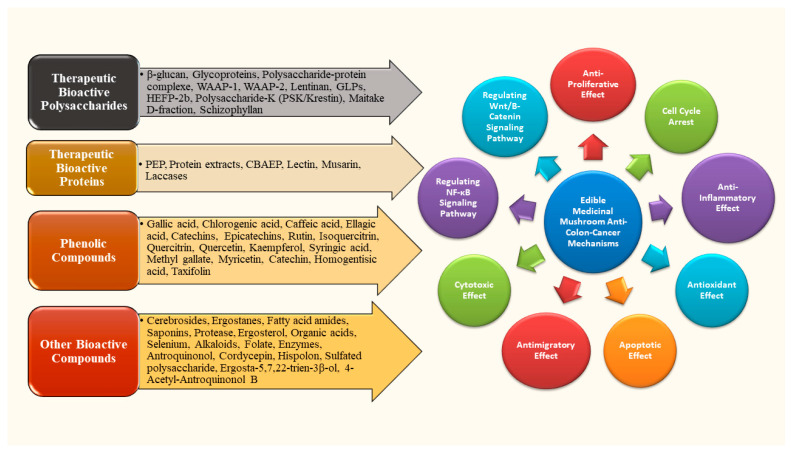
Anti-colon cancer mechanisms of edible and medicinal mushrooms. CBAEP—Cibacron blue affinity eluted protein; PEP—A novel bioactive protein in *Pleurotus eryngii*.

Edible and medicinal mushrooms may exert their anticancer effects through immunomodulation, antioxidation, and direct cytotoxicity in cancer cells [82]. Medicinal mushrooms have been recognized as potential agents to fight against cancer. Bioactive compounds in medicinal mushrooms interfere with the cellular pathways of cancer cells and stimulate the immune system to fight against cancer cells [83,84]. Figure 2 illustrates the mechanisms of action of edible mushrooms in colon cancer management.

### 3.1. Anti-Colon Cancer Mechanism Through Anti-Proliferative Effects

#### 3.1.1. Introduction to Anti-Proliferative Activity of Medicinal Mushrooms

Medicinal mushroom extracts and their bioactive compounds that exhibit anti-proliferative effects are emerging as alternative and complementary therapeutic strategies for treating colon cancer. Further research is required to optimize their bioactive component bioavailability and explain their molecular mechanisms.

**Figure 3:**
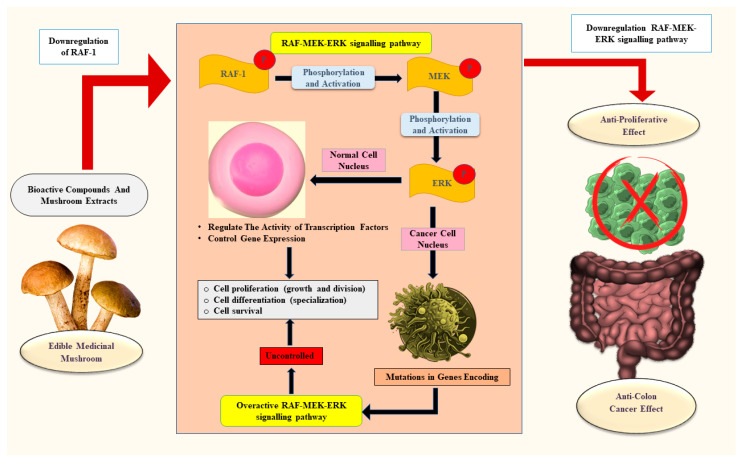
Anti-proliferative effects of edible and medicinal mushrooms against colon cancer. ERK—Extracellular signal-regulated kinase; MEK—Mitogen-activated protein kinase; RAF-1—RAF proto-oncogene serine/threonine-protein kinase.

Figure 3 illustrates the anti-proliferative effects of edible medicinal mushrooms against colon cancer. Several in vivo as well as in vitro studies further confirmed the anti-proliferative effects of edible medicinal mushrooms.

#### 3.1.2. In Vitro and In Vivo Studies of the Anti-Proliferative Activity of Medicinal Mushrooms

Aqueous extracts of *Auricularia polytricha*, *Macrolepiota procera*, and *Pleurotus ostreatus* showed an irreversible anti-proliferative effect against human colorectal adenocarcinoma cell line 205 (COLO-205) [19]. A study conducted on selenium-enriched *Pleurotus ostreatus* edible MM extract (SME) downregulated the expression of Raf-1 [20]. Raf-1 is a crucial protein kinase in the mitogen-activated protein kinase (MAPK) pathway that promotes growth, differentiation, and survival of cells. Due to gene mutation or under a constitutively active state of Raf-1, oncogenesis occurs, transforming normal cells into cancerous cells and increasing cancer cell proliferation in the colon [85]. The inhibition of colon cancer cell proliferation by Se-enriched mushrooms showed a dose-dependent nature. Purified selenomethionine mainly targeted the blocking of the RAF-MEK-ERK signaling pathway, which is involved in cell survival and proliferation [20]. Caproic acid, a medium-chain fatty acid found in *D. indusiata* edible MM, shows anti-proliferative effects against human colorectal [30]. Research conducted on the structural characterization and anti-colon cancer activity of *Agaricus bisporus* used a sequential extraction method (room temperature water, hot water, high-pressure hot water, dilute alkaline solution, and concentrated alkaline solution) to obtain five crude polysaccharides, and a homogeneous polysaccharide called ‘WAAP-1’ was purified from them using a DEAE Cellulose-52 column. WAAP-1 showed promising anticancer properties by significantly inhibiting the proliferation of HT-29 colon cancer cells [17].

A polyphenol-rich extract isolated from *Pleurotus eryngii* showed dose and time-dependent suppression of HCT-116 cell proliferation. The extract showed no inhibitory effect against normal human colonic myofibroblasts (CCD-18Co cells) [28]. PEP in *Pleurotus eryngii* showed dose and time-dependent suppression of HCT-116 and MC38 murine colon adenocarcinoma cell line [58]. The *α*-glucan fraction of *Pleurotus ostreatus* significantly decreased the growth of HT-29 cells in a dose-dependent manner [44]. A significant decrease in the number of precancerous lesions (aberrant crypt foci and microadenomas) was observed in mice fed the polysaccharide extract of *Pleurotus pulmonarius* fruiting bodies or mycelia. The reduction in precancerous lesions was due to decreased cell proliferation, increased cell death, and reduced levels of the inflammatory cytokine, tumor necrosis factor alpha (TNF-α) [86]. Aqueous extracts of *Auricularia polytricha*, *Macrolepiota procera*, and *Pleurotus ostreatus* demonstrated significant inhibition of the growth and proliferation of human colorectal adenocarcinoma cell line COLO-205 [19]. The methanolic extract of *Lignosus rhinocerotis* (Tiger milk mushroom) demonstrated strong anti-proliferative activity against HCT-116 cells, while the aqueous extract demonstrated weaker anti-proliferative activity. The IC_50_ values of the methanolic and aqueous extracts were 600 µg/mL and 1200 µg/mL, respectively [87]. *Boletus edulis* extracts showed dose-dependent and time-dependent reduction of Caco-2 cell viability. The IC_50_ values of Caco-2 cells at 24 h, 48 h, and 72 h incubation periods were >2000 μg/mL, 1880 ± 18 μg/mL, and 1509 ± 43 μg/mL, respectively, which demonstrated a significant anti-proliferative effect against human colon cancer cells [46].

#### 3.1.3. Limitations and Future Directions of In Vitro and In Vivo Studies on the Anti-Proliferative Activity of Medicinal Mushrooms

Many studies are limited to in vitro and animal studies and suggest that bioactive compounds inhibit proliferation by targeting oncogenic pathways. They are not clinically validated, which raises a critical issue about translatability to humans. Furthermore, the bioavailability of these bioactive compounds remains a major hurdle. Many mushroom-derived polysaccharides and proteins demonstrate poor absorption and raise concerns related to high doses that may risk toxicity. The standardization of extraction methods is another critical issue. Using different extracts raises concerns about varied concentrations and the types of bioactive compounds extracted. Future research must prioritize human trials to determine the anti-colon cancer mechanism through anti-proliferative effects. In addition, optimizing delivery systems through nanoencapsulation and exploring synergistic combinations with chemotherapy could improve the anti-proliferative effects against colon cancer cells.

### 3.2. Anti-Colon Cancer Mechanism Through Cell Cycle Arrest

#### 3.2.1. Introduction to Anti-Colon Cancer Mechanism Through Cell Cycle Arrest by Medicinal Mushrooms

Medicinal mushrooms demonstrate an anti-colon cancer mechanism through cell cycle arrest. The induction of cell cycle arrest in colon cancer cells is emerging as a promising approach due to the inability to maintain cellular homeostasis in cancer cells. Through various in vivo and in vitro studies, polysaccharides, polyphenols, proteins, and other bioactive compounds have demonstrated selective targeting of cancer cells by arresting their cell cycle progression.

#### 3.2.2. In Vitro and In Vivo Studies on Cell Cycle Arrest by Medicinal Mushrooms

**Figure 4:**
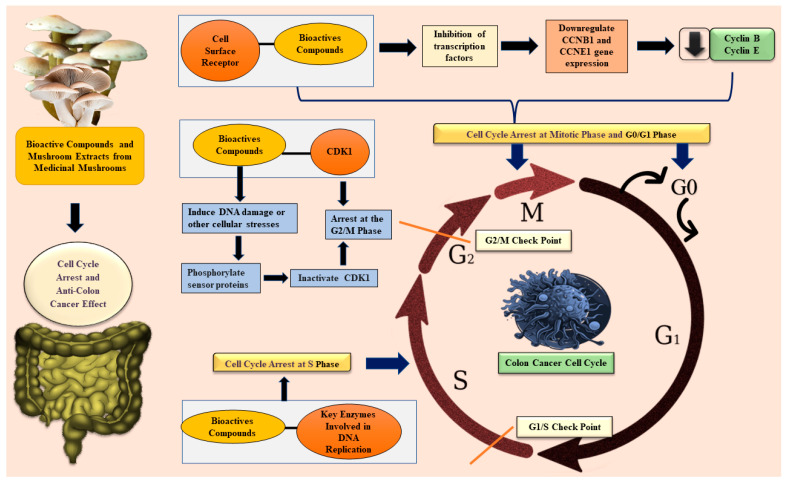
Cell cycle arrest induced by edible and medicinal mushrooms in colon cancer. CCNB1—Cyclin B1; CCNE1—Cyclin E1; CDK1—Cyclin-dependent kinase 1; DNA—Deoxyribonucleic acid.

Figure 4 illustrates the cell cycle arrest mechanism of edible medicinal mushrooms in colon cancer. A polyphenol-rich extract isolated from edible *Pleurotus eryngii* induced cell cycle arrest in HCT-116 cells. The expression of cyclin B and cyclin E, which are essential proteins for cell cycle progression, was downregulated in the mitotic phase of the cancer cell cycle [28]. PEP in *Pleurotus eryngii* downregulates cyclin B, cyclin E, and Cyclin-dependent kinase (CDC-2) proteins and arrests the cell cycle, leading to apoptosis induction [58]. The *Lentinus edodes* alcohol precipitate (LAP) obtained from *L. edodes* caused cell cycle arrest at the G0/G1 phase and prevented COLO 205 xenografted cancer cell proliferation in nude mice. Only 1 mg/mL of LAP was sufficient to arrest 61.3% of COLO 205 cancer cells in the G0/G1 phase of the cell cycle [21]. BSGLWE significantly induced cell cycle arrest at the G2/M phase in HCT-116 cells. The arrested cell percentages at 5 mg/mL (23.7%) and 7.5 mg/mL (32.2%) were higher when compared with the control (12.0%). After BSGLWE treatment, the proportion of HCT-116 cells in the G0/G1 phase slightly decreased, and the proportion of cancer cells in the S phase was significantly decreased. The G2/M checkpoint was confirmed by measuring cyclin B1 and cyclin A2. The mRNA levels of cyclin B1 and cyclin A were significantly reduced while the mRNA level of cell cycle arrest protein cyclin-dependent kinase inhibitor 1 (p21) was increased [31]. HEFP-2b polysaccharide arrested the colon cancer cell cycle at its S phase and inhibited HCT-116 and DLD1 cell growth [25]. The anti-proliferative activity of SLNT against HT-29 cells was concentration-dependent. SLNT at a 1600 μg/mL concentration demonstrated the strongest activity against HT-29 cells and the cell viability was 35.5% [32]. The extract of *Pleurotus sajor-caju* prevented further dividing and multiplying of cancer cells by inducing cell cycle arrest in HCT-116 cells at the G2/M phase. In addition, the extract interfered with the p21/ tumor protein p53 (p53) cell cycle regulation pathway [4]. The extract of *Boletus edulis* interfered with the G0/G1 phase and reduced the proportion of cancer cells in the S phase [46].

### 3.3. Anti-Colon Cancer Mechanism Through Anti-Inflammatory Effects

Chronic inflammation, including prolonged exposure to pro-inflammatory cytokines, is a risk factor for colorectal cancer (CRC). Bioactive compounds in medicinal mushrooms suppress inflammation-related signaling pathways such as NF-κB, Wnt/β-catenin, prostaglandin E2 (PGE2), and COX-2. Further clinical research is required to capture their full potential in human oncology.

**Figure 5:**
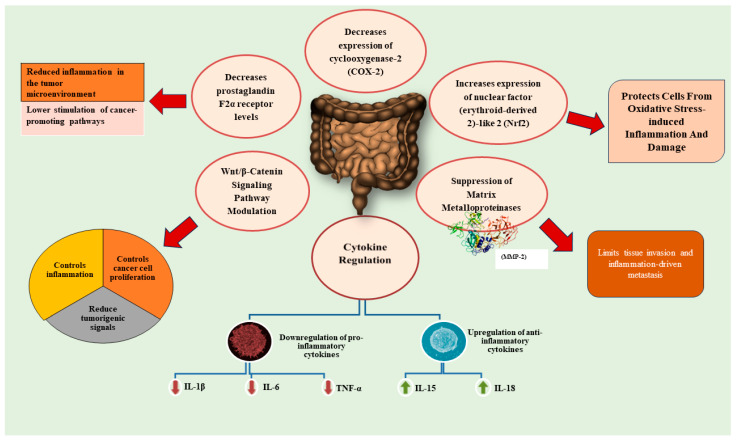
Anti-inflammatory effects of edible and medicinal mushrooms against colon cancer. COX-2—Cyclooxygenase-2; IL—Interleukin; MMP-2—Matrix metallopeptidase-2; Nrf2—Nuclear factor erythroid 2; TNF-α—Tumor necrosis factor alpha.

Figure 5 illustrates the anti-inflammatory effects of edible medicinal mushrooms against colon cancer. Polysaccharides purified from *Gloeostereum incarnatum* (GIPS) (30 mg/kg) significantly changed 89 of these cytokines (over 1.5-fold change). These altered cytokines were linked with inflammatory pathways, including the Wnt signaling pathway. Pro-inflammatory cytokines IL-1*β*, IL-6, and TNF-α were downregulated while IL-15 and IL-18 were promoted. GIPS suppressed matrix metallopeptidase-2 (MMP-2) enzyme [43,88]. The six families of interleukins (IL) (IL-1, -2, -6, -8, -10, -17) have their own ways of being involved in oncogenic or antitumor functions in colorectal cancer [89]. For instance, IL-1*β* promotes colon cancer cell proliferation and tumorigenesis and alters the tumor microenvironment [89,90,91].

IL-6 promotes mitosis, proliferation, metastasis, migration, and angiogenesis and provides a microenvironment suitable for the metastasis of cancer cells [89]. TNF-α is associated with CRC as it promotes tumor growth, invasion, and metastasis [92,93]. IL-15 inhibits cancer cell proliferation and angiogenesis and promotes apoptosis [89,94,95]. IL-18 improves intestinal barrier integrity and improves the immune system by acting on NK cells [89,91,96]. A study investigated the anti-inflammatory effects of the extract of *Agaricus bisporus* against Caco-2 cells. The Caco-2 cells were stimulated with LPS and TNF-α. The extract decreased the cyclooxygenase-2 and prostaglandin F2α receptor levels in cells associated with inflammation and increased the expression of nuclear factor (erythroid-derived 2)-like 2 associated with cellular protection. The anti-inflammatory activity was further supported by reducing the production of IL-6 in Caco-2 cells [97].

### 3.4. Anti-Colon Cancer Mechanism Through Antioxidant Effects

During energy production in cells, free radicals (FR) and oxidants are formed, which are either reactive oxygen species (ROS) or reactive nitrogen species (RNS). The excess production of these FRs and oxidants could cause oxidative stress and an imbalance in the antioxidant system. This causes detrimental effects, including colon cancer [30,98,99,100]. Some edible MM species are rich sources of antioxidants [19]. The methanolic extracts of five different medicinal mushrooms (*Phellinus linteus*, *Cordyceps sinensis*, *L. edodes*, *Coprinus comatus*, and *G. lucidum)* caused a prooxidant/antioxidant imbalance in colon cancer cell lines, leading to oxidative stress. *Phellinus linteus* showed the highest phenolic content, flavonoid content, and DPPH scavenging capacity. It was determined that anti-migratory effects had a positive correlation with increased superoxide anion radical production (O_2_^•−^) [18].

### 3.5. Anti-Colon Cancer Mechanism Through the NF-κB Signaling Pathway

NF-κB is a protein complex signaling pathway that may control the transcription of DNA, cytokine production, cell survival, and, in particular, inflammatory responses, cellular growth, and apoptosis, and it may be related to diseases such as cancer [101,102,103]. The activation of NF-κB in inflammatory bowel disease might induce cellular transformation, mediate cellular proliferation, and prevent the elimination of pre-neoplastic and fully malignant cells by upregulating anti-apoptosis proteins, increasing the risk of colorectal cancer [104]. However, NF-κB also may make a remarkable contribution to colon cancer progression through the regulation of the target genes that help in cell proliferation (Cyclin D1), angiogenesis (vascular endothelial growth factor (VEGF), IL-8, COX2), and metastasis (MMP9). It has been found that various edible and medicinal mushroom compounds have anti-colon cancer activity, which may help in cell proliferation, angiogenesis, and metastasis.

### 3.6. Anti-Colon Cancer Mechanism Through the Wnt/β-Catenin Signaling Pathway

The anti-colon cancer effects of GIPS were demonstrated in a mouse model (*Apc*^MinC^/*Gpt* mice), where the growth, size, and number of tumors were reduced after an 8-week administration but body weight was not affected. GIPS (90 mg/kg) showed better results than GIPS (30 mg/kg). GIPS regulated the Wnt/*β*-catenin signaling pathway, which promoted cancer cell proliferation, and showed resistance to chemotherapy. GIPS downregulated the expression of *β*-catenin, Frizzled-7, WNT1, LRP5/6, MMP-2, and MMP-9 and upregulated the expression of DKK1 and Kremen-2. In addition, GIPS suppressed GSK-3β phosphorylation in colon cancer tissues [26]. Abnormal activation of the Wnt/β-catenin signaling pathway is one of the main features in CRC. This occurs due to the mutation of either the APC gene (Adenomatous Polyposis Coli) or the β-catenin gene. The Wnt/β-catenin signaling pathway has significant roles in maintaining stem cell homeostasis and intestinal function in the human body. Mutations in APC or the β-catenin gene promote the accumulation of β-catenin in the cell nucleus, which eventually promotes colon cancer cell proliferation and tumorigenesis. Therefore, targeting this pathway to treat colon cancer would be an effective strategy [105,106]. MMP-1 gene expression is also associated with colon cancer cell invasion and migration [107,108].

### 3.7. Anti-Colon Cancer Mechanism Through Apoptotic Effects

#### 3.7.1. Initiation of Apoptotic Effects Through Intrinsic and Extrinsic Pathways

Table 1 illustrates the anti-colon cancer mechanism of edible medicinal mushrooms through apoptotic effects. Apoptosis is a well-regulated process that programs the death of cells. During colon cancer, the apoptotic pathway is dysregulated or inhibited through various mechanisms by the overexpression of antiapoptotic proteins or underexpression of proapoptotic proteins [109].The extrinsic pathway (death receptor-mediated) and intrinsic pathway (mitochondria-dependent) are the two primary pathways involved in initiating apoptosis [110].

#### 3.7.2. Apoptotic Effects of *Inonotus obliquus*, *Pleurotus ostreatus*, and *Agaricus bisporus*

Edible medicinal mushrooms have shown promising results against CRC by activating apoptotic pathways. IOWE inhibited the proliferation of HT-29 cells through apoptotic effects. IOWE decreased the level of Bcl-2, an anti-apoptotic protein, and increased the levels of Bcl-2-associated x (BAX), an apoptosis regulator, and caspase-3, triggering apoptosis [6]. Members of the Bcl-2 family of proteins are major contributors in CRC through colon cancer cell initiation, progression, and resistance to cancer treatments [111].

SME enhances the regulation of p53 and caspase-3 [20]. p53 protein regulates apoptosis. Notably, 60% of CRC patients have a mutation in this gene and show resistance to cancer treatments due to dysregulation of the apoptotic pathway [112]. Caspase-3 is a key enzyme involved in apoptosis that causes the death of cancer cells and acts as a tumor suppressor. However, recent studies have focused on the non-apoptotic function of caspase-3, which could contribute to tumor progression in the colon [113]. The α-glucan fraction of *Pleurotus ostreatus* induced apoptosis in HT-29 cells by increasing levels of BAX and cytosolic cytochrome-c proteins [44]. 

WAAP-1 promoted apoptosis of colon cancer cell line HT-29 and inhibited the epithelial-mesenchymal transition in HT-29 cells. The expression of caspase-3, BAX, and E-cadherin proteins was upregulated while Bcl-2 and Vimentin protein expression was downregulated [17]. WAAP-2 showed an apoptosis-inducing effect by arresting the HT-29 cell cycle at the S phase. Also, by upregulating caspase-3 and BAX protein expression and downregulating Bcl-2 protein expression, WAAP-2 promoted apoptosis. WAAP-2 induced the expression of E-cadherin and inhibited Vimentin expression. By inducing the expression of E-cadherin, the migration and invasion of colorectal cancer cells can be inhibited. By inhibiting Vimentin expression, the epithelial-mesenchymal transition can be affected, decreasing the invasive potential of CRC cells [42]. Natural killer cells treated with microencapsulated polysaccharide extract from *Agaricus bisporus* induced apoptosis in Caco-2 cells by downregulating the expression of genes that promote cell survival, such as Bcl-2 and transforming growth factor beta (TGF-*β)*, and upregulating NF-kappa-B inhibitor alpha (IkappaB-α) gene expression, which inhibits the NF-κB signaling pathway [40]. 

#### 3.7.3. Apoptotic Effects of *Pleurotus eryngii* and *Ganoderma lucidum*

A polyphenol-rich extract of *Pleurotus eryngii* caused apoptosis induction in HCT-116 cells. The molecular mechanisms involved downregulating cell cycle proteins and upregulating apoptosis proteins. The polyphenol extract upregulated caspase-3 and cleaved caspase-3 proteins, leading to cell death [28]. PEP upregulated apoptosis proteins such as p53 and c-poly (adp-ribose) polymerase (PARP), initiating cell death. The in vivo study conducted on PEP treatment of mice with transplanted colon cancer cells showed increasing levels of p21, p53, caspase-3, and cleaved caspase-3 in tumor tissues and suppressed tumor growth in mice. Increased cleavage of caspase-8 was observed, confirming the activation of the extrinsic apoptotic pathway. The activated caspase-8 then cleaved caspase-3 and PARP, ultimately leading to programmed cell death [58].

Apoptosis increased in colon cancer cells in a dose-dependent and time-dependent manner after BSGLWE treatment. BSGLWE treatment induced nuclear fragmentation, condensation, coagulation, and formation of apoptotic bodies in HCT-116 cells. BSGLWE upregulated survivin expression while downregulating Bcl-2 expression. In addition, Bcl-2, pro-caspase-3, pro-caspase-9, and cleaved PARP protein levels were altered. BSGLWE also induced death receptor-mediated apoptosis in HT-29 and HCT-116 cells through the activation and upregulation of caspase-8. Caspase-8 is a key initiator of the extrinsic apoptosis pathway. Subsequently, the upregulation of caspase-8 resulted in the cleavage and activation of caspase-3. This triggered programmed cell death in colon cancer cells. The expression of nonsteroidal anti-inflammatory drug-activated gene-1 (NAG-1) at both mRNA and protein levels was induced. Higher concentrations of BSGLWE (both 5 and 7.5 mg/mL) increased the secretion of NAG-1 into the cell culture medium [31]. NAG-1 induces various anticancer effects and its role in apoptosis is complex. NAG-1 acts as a tumor suppressor that inhibits cancer cell growth and promotes apoptosis. NAG-1 combined with the downregulation of Bcl-2 or related anti-apoptotic proteins enhances the apoptosis mechanism [114,115].

#### 3.7.4. Apoptotic Effects of *Lentinus edodes* as a Promising Medicinal Mushroom

The combined therapeutic effect of *Lentinus edodes* mycelium extract (LEM) and 5-fluorouracil against human colon cancer cells in xenografted nude mice showed promising antitumor activity by inducing apoptosis. Many COLO 205 cancer cells underwent apoptosis when treated with concentrations greater than 15 mg/mL LAP for 15 h. At the same time and concentration, COLO 205 cells showed morphological changes and internucleosomal DNA degradation. Also, LAP enhanced the effectiveness of 5-fluorouracil. The synergistic effect of LEM and 5-fluorouracil increased the expression of cell cycle inhibitor proteins p53, p21/Cip1, and cyclin-dependent kinase inhibitor 1B (p27/Kip1) and decreased the expression of cyclins B and D3 in tumor cells [21]. 

SLNT affects tumor growth and induces apoptosis in a dose-dependent manner. SLNT at 1600 μg/mL resulted in 48.50% apoptotic cells, while the control group’s value was 7.31%. AO/EB staining showed more apoptotic cells than normal cells. In DAPI staining, many SLNT-treated cells showed condensed chromatin, confirming the presence of apoptotic cells. SLNT treatment significantly reduced tumor size and weight. This mechanism was dose-dependent (0.2, 1.0, and 5.0 mg/kg inhibition percentages were 17.88%, 48.87%, and 57.90%, respectively), but the rates were lower than the positive control drug 5-FU (67.23% inhibition). SLNT activates mitochondrial apoptotic pathways, activates caspase-3, releases cytochrome c, and causes loss of mitochondrial membrane potential.

SLNT significantly increased the levels of TNF-α, a pro-inflammatory cytokine, and caspase-8 in HT-29 cells. TNF-α triggers apoptosis through an extrinsic pathway. Caspase-8 is one of the key components of the extrinsic pathway and acts as an initiator caspase. The SLNT-induced apoptosis was dependent on capase-8 activation to some extent. This was further confirmed through using a caspase-8 inhibitor, which reduced the extent of apoptosis induced by SLNT. This suggested that caspase-8 played a crucial role in mediating the anti-colon cancer effects of the *L. edodes* polysaccharide [32].

#### 3.7.5. Apoptotic Effects of Other Promising Medicinal Mushrooms in Anti-Colon Cancer Therapy

The hexane and chloroform fractions of *G. neo-japonicum* exert their anti-colon cancer activity against colonic carcinoma cells through the induction of apoptosis [27]. Polysaccharides extracted from *Pleurotus pulmonarius* induced dose-dependent apoptosis in human colon cancer cells. The extract regulated Bcl-2, BAX, and cytochrome C protein expression [86]. Andosan™ induced apoptosis in Caco-2 cells. Andosan™-treated groups showed significant increases in the abundance of both early and late apoptotic cells compared to untreated controls. The control group, 1.0% Andosan™, and 5.0% Andosan™ treated groups, respectively, showed 5.7% ± 1.5%, 15.3% ± 2.1%, and 35.6% ± 4.5% in early apoptosis, while late apoptosis showed 7.3% ± 2.1%, 35.6% ± 4.5%, and 39.7% ± 7.6% [35]. Protein extracts of *Calvatia lilacina*, *Pleurotus ostreatus*, and *Volvariella volvacea* demonstrated apoptosis induction in SW480 cells. *Pleurotus ostreatus* increased ROS production, depletion of glutathione, and loss of mitochondrial membrane potential, while other extracts dramatically increased the proportion of SW480 cells in the sub-G1 phase. *Volvariella volvacea* demonstrated a stronger apoptotic effect, increasing the proportion of SubG1 cells from 1.9% to 97.8% [60]. The n-hexane fraction of Pleurotus sajor-caju (PSC-hex) induced cancer cell apoptosis by breaking down the mitochondrial membrane potential in cancer cells, ROS generation, increasing the expression of p53, BAX, and caspase-3 proteins, and decreasing the expression of Bcl-2 [4].

### 3.8. Anti-Colon Cancer Mechanism Through Antimigratory Effects

Table 2 summarizes the anti-colon cancer mechanism through antimigratory effects. The antimigratory effects of methanolic extracts of *Phellinus linteus*, *Cordyceps sinensis*, *Lentinus edodes*, *Coprinus comatus*, and *Ganoderma lucidum* mushroom varieties were determined using the Transwell assay and immunofluorescence staining of *β*-catenin. *Phellinus linteus* showed significant antimigratory effects against both HCT-116 and SW-480 colon cancer cell lines. At the same time, all of the other mushroom extracts had significant anti-migratory effects against only the HCT-116 colon cancer cell line. The antimigratory effects had a positive correlation with both increased superoxide anion radical production (O_2_^•−^) and reduced expression of *β*-catenin protein [18].

### 3.9. Anti-Colon Cancer Mechanism Through Cytotoxic Effecst

Table 2 summarizes the anti-colon cancer mechanism through cytotoxic effects. The ethanolic extract of *Macrolepiota procera* and aqueous extract of *Pleurotus ostreatus* showed significant cytotoxic effects against the COLO-205 cell line [19]. The n-hexane extracts of *Hericium erinaceus*, *Metacordyceps neogunnii*, and *Dictyophora indusiata* MMs showed dose-dependent anticancer activity. *M. neogunnii* showed the highest cytotoxicity level (68.6 ± 3.6%) while *H. erinaceus* (18.3 ± 1.7%) and *D. indusiata* (19.3 ± 3.2%) demonstrated lower cytotoxicity levels against HCT-116 cells at 100 μg/mL [30]. The microcapsulated polysaccharide extract from *A. bisporus* significantly increased the CD16+CD56+ NK cell population, showing 74.09% cytotoxic activity against the Caco-2 cell line. Many treated cancer cells were arrested at the G0/G1 phase [40].

The Andosan™ concentration showed a strong inverse correlation with Caco-2 cell viability. Even the lowest concentration, 0.5%, showed a 14% reduction while the highest concentration, 5%, showed 90% cell viability reduction [35]. Protein extracts of *Calvatia lilacina*, *Pleurotus ostreatus*, and *Volvariella volvacea* demonstrated concentration-dependent cytotoxicity against SW480 and THP-1 cells. After 24 h of treatment with 500 μg/mL, all three protein extracts significantly decreased the cell viability of SW480 cells (8%, 2%, and 7%, respectively). *Pleurotus ostreatus* showed concentration-dependent cell viability reduction. The viability decreased to 39%, 10%, and 7% at 10 and 25 μg/mL, 50 μg/mL, and 100 μg/mL, respectively. *Volvariella volvacea* decreased cell viability by 20% at 10 μg/mL. The results suggested that the type of extract and the concentration used influence the degree of cytotoxicity [60]. PSC-hex demonstrated a strong cytotoxicity effect against colorectal cancer cells, with an IC_50_ value of 0.05 mg/mL [4]. Regardless of the tumor protein p53 status, the combined effect of LEM and 5-fluorouracil enhanced the cytotoxic effect. LAP demonstrated selective cytotoxicity against colon epithelial cells and less cytotoxicity against non-cancerous colon epithelial cells [21]. The hexane and chloroform fractions of *Ganoderma neo-japonicum* exerted their anti-colon cancer activity against colonic carcinoma cells through cytotoxic effects [27]. 

### 3.10. Anti-Colon Cancer Mechanism Through Gene Modulation

The bioactive compounds in medicinal mushrooms also exert their anti-colon cancer effects through gene modulation. This mainly involves critical genes such as Baculoviral IAP Repeat-Containing 5 (BIRC5), human telomerase reverse transcriptase (hTERT), hypoxia-inducible factor-1 alpha (HIF-1α or HIF1A), and Multidrug Resistance Protein 1 (MDR1). The following sections delve into the anti-colon cancer mechanism through gene modulation.

#### 3.10.1. Anti-Colon Cancer Mechanism Through BIRC5 (Survivin) Modulation

BIRC5, which is also known as survivin, plays a significant role as an inhibitor of the apoptosis protein (IAP) family and as an immune-related gene. It functions as an inhibitor of apoptosis (IAP) and regulates cell division by functioning as a chromosomal passenger protein. Overexpression of BIRC5 may show relationships with various types of cancer, particularly human colon cancer. BIRC5 interacts with other molecular pathways, such as Wnt/β-catenin and p53, and supports tumor growth and evasion of cell death [116,117]. 

Polysaccharides found in medicinal mushrooms such as *Ganoderma lucidum*, *Phellinus linteus*, and *Lentinus edodes* indirectly downregulate BIRC5 by inhibiting the NF-κB and PI3K/AKT/mTOR pathways and direct suppression at transcriptional and translational levels. Proteomic analyses conducted during research studies confirmed that mushroom extracts reduced BIRC5 levels in colon cancer cell lines. This was correlated with decreased cell proliferation and increased apoptosis. Triterpenoids present in *Ganoderma lucidum* suppressed BIRC5 expression through blocking STAT3 signaling, a protein-mediated pathway of gene expression that upregulates survivin expression. Due to this, the caspases were activated, leading to apoptosis in colon cancer cells. Lectins and proteins present in *Ganoderma lucidum* induced the p53-mediated suppression of BIRC5, promoting cancer cell cycle arrest and programmed cell death. Overall, downregulation of BIRC5 inhibits the colon cancer survival rate through inducing programmed cell death, restricting cancer cell proliferation, and enhancing chemo sensitivity [118,119,120]. A study conducted on the bioactive compounds present in a mixture of *Trametes versicolor*, *Ganoderma lucidum*, and *Dioscorea opposite* extract interfered with BIRC5 through proteomic and metabolic reprogramming. The mechanism of interference with BIRC5 mainly included the downregulation of BIRC5 and indirect suppression of BIRC5 through metabolic and translational reprogramming. Direct downregulation of BIRC5 was clearly demonstrated in the tandem mass tag (TMT) proteomics analysis. BIRC5 inhibition promotes apoptosis in cancer cells. BIRC5 production was indirectly reduced through disrupting key translation-related protein synthesis such as EIF4 family proteins. The mushroom extract also created an unfavorable environment for cancer cells and suppressed the anti-apoptotic mechanism. These studies suggested that the bioactive compounds in the mushroom extract downregulated BIRC5 and promoted the apoptosis of cancer cells [121]. 

#### 3.10.2. Anti-Colon Cancer Mechanism Through Telomerase Reverse Transcriptase Modulation

hTERT is the catalytic subunit of telomerase, which is responsible for maintaining the length of telomeres. This leads to shortening of telomeres during each cell division and eventually leads to apoptosis. However, in colon cancer cells, hTERT reactivates and allows proliferation. A study conducted on hTERT proteins in colorectal cancer investigated the expression and tumorigenesis activity of hTERT, which is more frequent in colorectal tumors. Certain clinicopathological features were also associated with hTERT expression. Immunohistochemistry showed that increased levels of hTERT were associated with tumor behavior in colon cancer [122,123]. A study conducted using the combined extract of both *G. lucidum* spores and *Sanghuangporus vaninii* demonstrated that TERT mediated the Wnt signaling pathway by downregulating hTERT [124]. A study conducted using *Ganoderma tsugae* and its fungal immunomodulatory protein (FIP) inhibited the activity of hTERT. Many studies have been conducted to investigate the effect of hTERT in human lung carcinoma cells rather than in human colon cancer cells. Therefore, more studies on colorectal cancer and hTERT should be conducted to identify and understand the mechanisms and specific bioactive compounds present in medicinal mushrooms. Fungal immunomodulatory proteins transcriptionally downregulated hTERT, leading to telomerase inhibition in the A549 human lung adenocarcinoma cell line [125]. 

#### 3.10.3. Anti-Colon Cancer Mechanism Through Hypoxia-Inducible Factor 1 Alpha Modulation

HIF-1α plays a significant role in the cellular response to low oxygen levels. It functions as a key transcriptional regulator. HIF-1α promotes angiogenesis, metabolic adaptation, and cell survival, leading to colon cancer aggressiveness and tumor growth in the human body through hypoxia-mediated pathways and under normoxic conditions. A study was conducted to investigate HIF-1α-driven colon cancer proliferation, demonstrating that overexpression of HIF-1α enhances tumor growth in colon cancer cells [126,127]. 

*G. lucidum* is a medicinal mushroom that has triterpenes and polysaccharides as bioactive compounds. These compounds exhibit antitumor activity under hypoxic conditions by inhibiting HIF-1α. *Antrodia camphorata* also suppresses HIF-1α expression and helps to reduce tumor hypoxia adaptation. The bioactive compounds in these mushrooms had the ability to interfere with tumor hypoxia signaling, which is a critical pathway in colon cancer progression [128]. *Ganoderma lucidum* (Lingzhi or Reishi) aqueous mushroom extract (GLE) plays a role on hypoxia-induced responses that include HIF-1α modulation. The results demonstrated that the extract dramatically reduced hypoxia-induced HIF-1α stabilization in rat thymocytes. Generally, HIF-1α levels increase under hypoxic conditions. However, GLE treatment suppressed this upregulation of HIF-1α and contributed to the restoration of redox balance and reduction of inflammatory responses [129]. 

#### 3.10.4. Anti-Colon Cancer Mechanism Through Multidrug Resistance Protein 1 Modulation

MDR1, also known as ABCB1 or P-glycoprotein, is an ATP-binding cassette (ABC) transporter. MDR1 functions as an efflux pump that expels chemotherapeutic agents out of cancer treated cells. MDR1 decreases intracellular drug accumulation and reduces the effectiveness of cancer treatments. Increased expression of MDR1 correlates with poor prognosis, and reduced response to therapy has become a major issue in treatments [130]. 

Mushroom-derived bioactive compounds downregulate MDR1 expression by inhibiting the NF-κB, AP-1, and PI3K/Akt pathways. These compounds can block MDR1 efflux activity and enhance intracellular drug accumulation. *G. lucidum* has triterpenoids such as ganoderic acids, which inhibit NF-κB and MAPK signaling and suppress MDR1. *T. versicolor* contains PSK and polysaccharide-peptide (PSP), which decrease MDR1-mediated resistance by inhibiting PI3K/Akt and immunomodulation. *Phellinus linteus* contains hispidin and polysaccharides that downregulate MDR1 via ROS-mediated pathways [78]. A study conducted on *G. lucidum* identified polysaccharides, triterpenes (ganoderic acids), and other metabolites as major bioactive components in *G. lucidum*. The bioactive compounds modulate the NF-κB and STAT3 signaling pathways. NF-κB is identified as a transcriptional regulator of MDR1 and, by suppressing the activation of NF-κB, indirectly downregulates the expression of MDR1, which may be very valuable for chemo treatments. MDR1 downregulation decreases the expression of pro-inflammatory cytokines because chronic inflammation promotes MDR1 upregulation [23]. Ganoderic acid, a bioactive triterpenoid derived from *G. lucidum*, regulated MDR1 expression in the surg pathol-derived colorectal cancer cell line 620 (SW620/Ad300) cells. These compounds inhibit NF-κB activation, which indirectly downregulates MDR1 expression [131]. 

## 4. Challenges and Limitations

Edible medicinal mushrooms in colon cancer therapy face several challenges and limitations, particularly, limited clinical evidence, variability in the composition of mushroom species, mushroom cultivation and preparation techniques, potential for drug interactions, quality control and standardization, limited understanding of mechanisms, potential side effects, and ethical considerations. While many edible medicinal mushrooms demonstrate anti-colon cancer properties, the specific mechanisms are yet to be discovered. Establishing consistent efficacy, quality, and safety is critical in mushroom-derived products. Well-designed clinical trials are crucial before using medicinal mushrooms in clinical practice. Mushroom-derived drug development and delivery systems require new modifications to improve their pharmacokinetics and therapeutic efficacy. It is necessary to develop a clear regulatory framework for both patients and healthcare providers and to address ethical concerns [36,132,133,134].

## 5. Future Directions and Research Opportunities

Globally, colon cancer has become one of the most significant causes of mortality. Conventional therapies for cancers have significant adverse impacts on patients and their limitations encourage the exploration of alternative therapies. Edible MMs have gained significant attention in recent years for their potent anticancer properties. Further research should be focused on mechanistic, translational, and clinical studies to capture their full potential to treat colon cancer. Research in this field should prioritize bioactive compound isolation, identifying their mechanisms of action, developing synergistic therapeutic effects, and standardizing delivery systems. MMs improve the quality of life of cancer patients by preventing lymph node metastasis and decreasing the side effects of chemotherapy [82]. It is important to continue the identification and characterization of different anti-colon cancer compounds in mushroom species and understand their mechanisms of action. A deeper understanding of how these bioactive compounds exert their effects is important to developing effective and safer anti-colon cancer therapeutics. Novel drugs can be formulated using these active compounds but they need to be confirmed through rigorous human clinical trials. Combining mushroom-derived compounds with conventional cancer treatments may offer a synergistic approach to fight against colon cancers [82,135,136]. 

As strong suggestions for future improvements in this field, AI-driven bioactive compound discovery and analysis could be used to predict synergistic mushroom compound combinations to treat colorectal cancer-specific pathways [137]. Engineered mushrooms through clustered regularly interspaced short palindromic repeats (CRISPR) gene editing technology can be used to develop mushroom species that can overexpress anti-CRC compounds [138]. Oral mushroom spore-based vaccines could be introduced as a new concept in vaccine development, where people with colorectal cancers could use spores to deliver CRC-specific antigens to the gut. Eventually this will stimulate an immune response in the gut to fight against colorectal cancer [139,140,141]. 

## 6. Conclusions

Colon cancer has become a major global health issue. The search for novel, affordable, and effective treatments with minimum side effects is demanding. This review comprehensively explores the anti-colon cancer properties of edible medicinal fungi. It is evident that bioactive compounds derived from medicinal mushroom species show significant effects against colon cancer. These natural compounds exert their actions through various mechanisms, demonstrating anti-proliferative, pro-apoptotic, and anti-metastatic effects in preclinical models. Also, a synergistic effect can be gained by combining medicinal mushrooms with standard chemotherapy or combining multiple mushroom species. While current research is promising, well-designed human clinical trials are crucial to confirm their effectiveness and safety. Improving extraction methods and formulations could further enhance their therapeutic benefits.

## Figures and Tables

**Table 1:** Anti-colon cancer mechanism of edible and medicinal mushroom through apoptotic effects.

Scientific Name	Common Name	Active Component/Fraction	Extraction Method	Experimental Model	Mechanism	References
*Agaricus bisporus*	White button mushroom	WAAP-1, WAAP-2	Polysaccharide extraction	HT-29 human colon cancer cells	Upregulation of Caspase-3, BAX, E-cadherin; downregulation of Bcl-2, Vimentin	[17,42]
		Microencapsulated polysaccharide extract	Polysaccharide extraction	Caco-2 human colon cancer cells	Downregulation of Bcl-2, TGF-β; upregulation of IkappaB-α; inhibits NF-κB signaling pathway	[40]
*Lentinus edodes*	Shiitake mushroom	LAP	Mycelium extract	COLO 205 human colon cancer cells	Upregulation of p53, p21/Cip1, p27/Kip1; downregulation of cyclins B and D3; induces apoptosis	[21]
		SLNT	Water extraction	HT-29 human colon cancer cells	Activation of mitochondrial apoptotic pathways, caspase-3 and caspase-8 activation, cytochrome c release, MMP loss	[32]
*Pleurotus eryngii*	King oyster mushroom	Polyphenol-rich extract, PEP (Protein)	Polyphenol extraction	HCT-116 human colon cancer cells	Upregulation of caspase-3, *caspase-8* p53, c-PARP; cleaves caspase-3; induces cell death	[28,58,59]
*Pleurotus ostreatus*	Oyster mushroom	α-Glucan	Polysaccharide extraction	HT-29 human colon cancer cells	Upregulation of BAX, cytochrome c; induces apoptosis	[44]
		Protein extract	Protein extraction	SW480 human colon cancer cells	Increased ROS production, glutathione depletion, loss of mitochondrial membrane potential	[60]
*Pleurotus pulmonarius*	Indian oyster mushroom	Polysaccharides	Fruiting body/mycelium extract	HT-29 human colon cancer cells	Regulation of Bcl-2, BAX, cytochrome C; Induces apoptosis	[86]
*Volvariella volvacea*	Straw mushroom	Protein extract	Protein extraction	SW480 human colon cancer cells	Increased SubG1 phase cells, loss of mitochondrial membrane potential	[60]
*Agaricus blazei, Hericeum erinaceus, Grifola frondosa*	-	Andosan™	Water extraction	Caco-2 human colon cancer cells	Induces early and late apoptosis	[35]
*Ganoderma neo-japonicum*	Reishi mushroom	Hexane and chloroform fractions	Fraction extraction	Colonic carcinoma cells	Induction of apoptosis	[27]
*Ganoderma lucidum*		BSGLWE	Water extraction	HCT-116 human colon cancer cells	Upregulation of NAG-1; downregulation of Bcl-2, pro-caspase-3, pro-caspase-9; cleaves PARP; caspase-8 activation	[31]
*Pleurotus sajor-caju*	-	PSC-hex	n-hexane extraction	-	ROS generation; p53, BAX, caspase-3 upregulation; Bcl-2 downregulation	[4]

Abbreviations: Andosan™—Water extracts of the mycelia of *Agaricus blazei* (82.4%), *Hericeum erinaceus* (14.7%), and *Grifola frondosa* (2.9%); BAX—Bcl-2-associated x, apoptosis regulator; Bcl-2—B-cell lymphoma 2; BSGLWE—Sporoderm-broken spores of *G. lucidum* water extract; Caco-2—Human colorectal adenocarcinoma cell line; COLO-205—Human colorectal adenocarcinoma cell line 205; HCT-116—Human colorectal carcinoma cell line 116; HT-29—Human colorectal adenocarcinoma cell line 29; IkappaB-α—NF-kappa-B inhibitor alpha; LAP—*Lentinus edodes* alcohol precipitate; NAG-1—Nonsteroidal anti-inflammatory drug-activated gene-1; NF-κB—Nuclear factor kappa-light-chain-enhancer of activated B cells; p21/Cip1 or CDKN1A—Cyclin-dependent kinase inhibitor 1; p27/Kip1 or CDKN1B—Cyclin-dependent kinase inhibitor 1B; p53—Tumor protein p53; ROS—Reactive oxygen species; PARP—Poly (ADP-ribose) polymerase; PSC-hex—n-hexane fraction of *Pleurotus sajor-caju*; SLNT—Water-extracted polysaccharide from *Lentinus edodes*; SW-480—Surg pathol-derived colorectal cancer cell line 480; TGF-β—Transforming growth factor beta; WAAP—A homogeneous neutral polysaccharide.

**Table 2:** **Table *2*.** Anti-colon cancer mechanism through antimigratory effects and cytotoxic effects.

Scientific Name	Common Name	Active Compound/Fraction	Experimental Model	Mechanism of Action	References
*Phellinus linteus*	N/A	Methanolic extract	HCT-116 and SW-480 colon cancer cell lines	Significant antimigratory effects against both cell lines; correlated with increased superoxide anion radical production and reduced β-catenin expression	[18]
*Cordyceps sinensis*	N/A	Methanolic extract	HCT-116 colon cancer cell line	Significant antimigratory effect; correlated with increased superoxide anion radical production and reduced β-catenin expression	[18]
*Lentinus edodes*	Shiitake mushroom	Methanolic extract	HCT-116 colon cancer cell line	Significant antimigratory effect; correlated with increased superoxide anion radical production and reduced β-catenin expression	[18]
*Coprinus comatus*	Shaggymane	Methanolic extract	HCT-116 colon cancer cell line	Significant antimigratory effect; correlated with increased superoxide anion radical production and reduced β-catenin expression	[18]
*Ganoderma lucidum*	Reishi mushroom	Methanolic extract	HCT-116 colon cancer cell line	Significant antimigratory effect; correlated with increased superoxide anion radical production and reduced β-catenin expression	[18]
*Macrolepiota procera*	Parasol mushroom	Ethanolic extract	COLO-205 human colon cancer cell line	Significant cytotoxic effect	[19]
*Pleurotus ostreatus*	Oyster mushroom	Aqueous extract	COLO-205 human colon cancer cell line	Significant cytotoxic effect	[19]
*Hericium erinaceus*	Lion’s mane mushroom	n-hexane extract	HCT-116 human colon carcinoma cell line	Lower cytotoxicity level	[30]
*Metacordyceps neogunnii*	N/A	n-hexane extract	HCT-116 human colon carcinoma cell line	Highest anti-colon cancer effect (68.6 ± 3.6% cytotoxicity)	[30]
*Dictyophora indusiata*	Bamboo mushroom	n-hexane extract	HCT-116 human colon carcinoma cell line	Lower cytotoxicity level	[30]
*Agaricus bisporus*	Button mushroom	Microcapsulated polysaccharide extract	Caco-2 human colon cancer cell line	Increased CD16+CD56+ NK cell population; 74.09% cytotoxic activity; G0/G1 phase cell cycle arrest	[40]
*Calvatia lilacina*	N/A	Protein extract	SW480 and THP-1 cells	Concentration-dependent cytotoxicity; significant decrease in cell viability at high concentrations	[60]
*Pleurotus ostreatus*	Oyster mushroom	Protein extract	SW480 cell line	Concentration-dependent cytotoxicity; significant decrease in cell viability at high concentrations	[60]
*Volvariella volvacea*	Straw mushroom	Protein extract	SW480 cell line	Concentration-dependent cytotoxicity; significant decrease in cell viability at high concentrations	[60]
*Ganoderma neo-japonicum*	N/A	Hexane and chloroform fractions	Colonic carcinoma cells	Cytotoxic effect	[27]
*Lentinus edodes*	Shiitake mushroom	Alcohol precipitate	Colon epithelial cells	Selective cytotoxicity in cancerous cells	[21]
*PSC-hex*	N/A	Hexane extract	Colorectal cancer cells	Strong cytotoxicity (IC50 = 0.05 mg/mL)	[4]
*Agaricus blazei, Hericeum erinaceus, Grifola frondosa*	-	Andosan™	Caco-2 human colon cancer cell line	Concentration-dependent cytotoxic effects; cell viability reduction correlated with concentration	[35]

Abbreviations: Andosan™—Water extracts of the mycelia of Agaricus blazei (82.4%), Hericeum erinaceus (14.7%) and Grifola frondosa (2.9%); Caco-2—Human colorectal adenocarcinoma cell line; COLO-205—Human colorectal adenocarcinoma cell line 205; HCT-116—Human colorectal carcinoma cell line 116; HT-29—human colorectal adenocarcinoma cell line 29; NK cells—Natural killer cells; SW-480—Surg pathol-derived colorectal cancer cell line 480; THP-1—Tohoku Hospital Pediatrics-1 human monocytic leukemia cell line.

## Abbreviations

The following abbreviations are used in this manuscript:

ABC	ATP-binding cassette
Andosan™	Water extracts of the mycelia of *Agaricus blazei* (82.4%), *Hericeum erinaceus* (14.7%), and *Grifola frondosa* (2.9%)
BAX	Bcl-2-associated x, apoptosis regulator
Bcl-2	B-cell lymphoma 2
BIRC5	Baculoviral IAP repeat-containing 5
BSGLWE	Sporoderm-broken spores of *G. lucidum* water extract
Caco-2	Human colorectal adenocarcinoma cell line
CBAEP	Cibacron blue affinity eluted protein
CCNB1	Cyclin B1
CCNE1	Cyclin E1
CDK1	Cyclin-dependent kinase 1
COLO-205	Human colorectal adenocarcinoma cell line 205
COX-2	Cyclooxygenase-2
CRC	Colorectal cancer
CRISPR	Clustered regularly interspaced short palindromic repeats
DNA	Deoxyribonucleic acid
ERK	Extracellular signal-regulated kinase
FIP	Fungal immunomodulatory protein
GIPS	Polysaccharides purified from *Gloeostereum incarnatum*
GLE	*Ganoderma Lucidum* extract
GLP	*Ganoderma lucidum* polysaccharides
GLSF	*Ganoderma lucidum* extract containg spores and fruiting bodies (30:8 ratio)
HCT-116	Human colorectal carcinoma cell line 116
HIF-1α or HIF1A	Hypoxia-inducible factor-1 alpha
HT-29	Human colorectal adenocarcinoma cell line 29
hTERT	Human telomerase reverse transcriptase
IkappaB-α	NF-kappa-B inhibitor alpha
IL	Interleukin
LAP	*Lentinus edodes* alcohol precipitate
*LEM*	*Lentinus edodes* mycelium extracts
MDR1	Multidrug resistance protein 1
MEK	Mitogen-activated protein kinase
MM	Medicinal mushrooms
MMP-2	Matrix metallopeptidase-2
MRP1	Multidrug resistance-associated protein 1
NAG-1	Nonsteroidal anti-inflammatory drug-activated gene-1
NF-κB	Nuclear factor kappa-light-chain-enhancer of activated B cells
NK cells	Natural killer cells
Nrf2	Nuclear factor erythroid 2
p21/Cip1 or CDKN1A	Cyclin-dependent kinase inhibitor 1
p27/Kip1 or CDKN1B	Cyclin-dependent kinase inhibitor 1B
p53	Tumor protein p53
PARP	Poly (ADP-ribose) polymerase
PSC-hex	n-hexane fraction of *Pleurotus sajor-caju*
RAF-1	RAF proto-oncogene serine/threonine-protein kinase
ROS	Reactive oxygen species
SLNT	Water-extracted polysaccharide from *Lentinus edodes*
SW-480	Surg pathol-derived colorectal cancer cell line 480
SW620/Ad300	Surg pathol-derived colorectal cancer cell line 620
TGF-β	Transforming growth factor beta
THP-1	Tohoku Hospital Pediatrics-1 human monocytic leukemia cell line
TNF-α	Tumor necrosis factor alpha
WAAP	A homogeneous neutral polysaccharide

## References

[1] Chaturvedi V.K., Agarwal S., Gupta K.K., Ramteke P.W., Singh M.P. (2018). Medicinal mushroom: Boon for therapeutic applications. 3 Biotech..

[2] Valverde M.E., Hernández-Pérez T., Paredes-López O. (2015). Edible mushrooms: Improving human health and promoting quality life. Int. J. Microbiol..

[3] Chang S.T., Wasser S.P. (2012). The role of culinary-medicinal mushrooms on human welfare with a pyramid model for human health. Int. J. Med. Mushrooms..

[4] Finimundy T.C., Abreu R.M.V., Bonetto N., Scariot F.J., Dillon A.J.P., Echeverrigaray S., Barros L., Ferreira I.C.F.R., Henriques J.A.P., Roesch-Ely M. (2018). Apoptosis induction by *Pleurotus sajor-caju* (Fr.) Singer extracts on colorectal cancer cell lines. Food Chem. Toxicol..

[5] Hu D.D., Zhang R.Y., Zhang G.Q., Wang H.X., Ng T.B. (2011). A laccase with antiproliferative activity against tumor cells from an edible mushroom, white common Agrocybe cylindracea. Phytomedicine.

[6] Lee S.H., Hwang H.S., Yun J.W. (2009). Antitumor activity of water extract of a mushroom, *Inonotus obliquus*, against HT-29 human colon cancer cells. Phytother. Res..

[7] Torre L.A., Siegel R.L., Ward E.M., Jemal A. (2016). Global cancer incidence and mortality rates and trends--an update. Cancer Epidemiol. Biomark. Prev..

[8] Rawla P., Sunkara T., Barsouk A. (2019). Epidemiology of colorectal cancer: Incidence, mortality, survival, and risk factors. Przeglad Gastroenterologiczny..

[9] Singha K., Hor P.K., Soren J.P., Mondal J., Mondal K.C., Pati B.R., Mohapatra P.K.D. (2021). Exploration of bioactive prospects of a polysaccharide fraction from *Termitomyces heimii* against colorectal cancer and broad-spectrum bacteria. Bioact. Carbohydr. Diet. Fibre.

[10] Abdel-Azeem A.M., Abdel-Azeem M.A., Khalil W.F. (2019). Endophytic fungi as a new source of antirheumatoid metabolites. Bioactive Food as Dietary Interventions for Arthritis and Related Inflammatory Diseases.

[11] Hamza A., Mylarapu A., Krishna K.V., Kumar D.S. (2024). An insight into the nutritional and medicinal value of edible mushrooms: A natural treasury for human health. J. Biotechnol..

[12] Frljak J., Mulabećirović A.H., Isaković S., Karahmet E., Toroman A., Frljak J., Mulabećirović A.H., Isaković S., Karahmet E., Toroman A. (2021). Biological active components of selected medical fungi. Open J. Prev. Med..

[13] Kumar K., Mehra R., Guiné R.P.F., Lima M.J., Kumar N., Kaushik R., Ahmed N., Yadav A.N., Kumar H. (2021). Edible mushrooms: A comprehensive review on bioactive compounds with health benefits and processing aspects. Foods.

[14] Khatun S., Islam A., Cakilcioglu U., Chatterjee N.C. (2012). Research on mushroom as a potential source of nutraceuticals: A review on Indian perspective. J. Exp. Agric Int..

[15] He Z., Lin J., He Y., Liu S. (2022). Polysaccharide-peptide from *Trametes versicolor*: The potential medicine for colorectal cancer treatment. Biomedicines.

[16] Sadowska A., Zapora E., Sawicka D., Niemirowicz-Laskowska K., Suraży ński A., Sułkowska-Ziaja K., Kała K., Stocki M., Wołkowycki M., Bakier S. (2020). *Heterobasidion annosum* induces apoptosis in DLD-1 cells and decreases colon cancer growth in in vivo model. Int. J. Mol. Sci..

[17] Zhang N., Liu Y., Tang F.Y., Yang L.Y., Wang J.H. (2023). Structural characterization and in vitro anti-colon cancer activity of a homogeneous polysaccharide from *Agaricus bisporus*. Int. J. Biol. Macromol..

[18] Šeklić D.S., Stanković M.S., Milutinović M.G., Topuzović M.D., Štajn A.Š., Marković S.D. (2016). Cytotoxic, antimigratory, pro-and antioxidative activities of extracts from medicinal mushrooms on colon cancer cell lines. Arch. Biol. Sci..

[19] Arora S., Goyal S., Balani J., Tandon S. (2013). Enhanced antiproliferative effects of aqueous extracts of some medicinal mushrooms on colon cancer cells. Int. J. Med. Mushrooms.

[20] Fekry T., Salem M.F., Abd-Elaziz A.A., Muawia S., Naguib Y.M., Khalil H. (2022). Anticancer properties of selenium-enriched oyster culinary-medicinal mushroom, *Pleurotus ostreatus* (Agaricomycetes), in colon cancer in vitro. Int. J. Med. Mushrooms.

[21] Wu C.H., Wu C.C., Ho Y.S. (2007). Antitumor activity of combination treatment of *Lentinus edodes* mycelium extracts with 5-Fluorouracil against human colon cancer cells xenografted in nude mice. J. Cancer Mol..

[22] Liu N., Zou S., Xie C., Meng Y., Xu X. (2023). Effect of the β-glucan from *Lentinus edodes* on colitis-associated colorectal cancer and gut microbiota. Carbohydr. Polym..

[23] Liu M.M., Liu T., Yeung S., Wang Z., Andresen B., Parsa C., Orlando R., Zhou B., Wu W., Li X. (2021). Inhibitory activity of medicinal mushroom *Ganoderma lucidum* on colorectal cancer by attenuating inflammation. Precis. Clin. Med..

[24] Wachtel-Galor S., Yuen J., Buswell J.A., Benzie I.F.F. (2011). Ganoderma lucidum (Lingzhi or Reishi). Herbal Medicine: Biomolecular and Clinical Aspects.

[25] Liu J.Y., Hou X.X., Li Z.Y., Shan S.H., Chang M.C., Feng C.P., Wei Y. (2020). Isolation and structural characterization of a novel polysaccharide from *Hericium erinaceus* fruiting bodies and its arrest of the cell cycle at S-phage in colon cancer cells. Int. J. Biol. Macromol..

[26] He J., Yang A., Zhao X., Liu Y., Liu S., Wang D. (2021). Anti-colon cancer activity of water-soluble polysaccharides extracted from *Gloeostereum incarnatum* via Wnt/β-catenin signaling pathway. Food Sci. Hum. Well..

[27] Lau M.F., Chua K.H., Sabaratnam V., Kuppusamy U.R. (2021). In vitro and in silico anticancer evaluation of a medicinal mushroom, *Ganoderma neo-japonicum* Imazeki, against human colonic carcinoma cells. Biotech. Appl. Biochem..

[28] Hu Q., Yuan B., Xiao H., Zhao L., Wu X., Rakariyatham K., Zhong L., Han Y., Muinde Kimatu B., Yang W. (2018). Polyphenols-rich extract from *Pleurotus eryngii* with growth inhibitory of HCT116 colon cancer cells and anti-inflammatory function in RAW264.7 cells. Food Funct..

[29] Peng H., Shahidi F. (2020). Bioactive compounds and bioactive properties of Chaga (*Inonotus obliquus*) mushroom: A review. J. Food Bioact..

[30] Daba G.M., Elkhateeb W.A., El-Dein A.N., Ahmed E.F., El Hagrassi A.M., Fayad W., Wen T.C. (2020). Therapeutic potentials of n-hexane extracts of the three medicinal mushrooms regarding their anti-colon cancer, antioxidant, and hypocholesterolemic capabilities. Biodiversitas.

[31] Na K., Li K., Sang T., Wu K., Wang Y., Wang X. (2017). Anticarcinogenic effects of water extract of sporoderm-broken spores of *Ganoderma lucidum* on colorectal cancer in vitro and in vivo. Int. J. Oncol..

[32] Wang J., Li W., Huang X., Liu Y., Li Q., Zheng Z., Wang K. (2017). A polysaccharide from *Lentinus edodes* inhibits human colon cancer cell proliferation and suppresses tumor growth in athymic nude mice. Oncotarget.

[33] Bisen P.S., Baghel R.K., Sanodiya B.S., Thakur G.S., Prasad G.B.K.S. (2010). *Lentinus edodes*: A macrofungus with pharmacological activities. Curr. Med. Chem..

[34] Firenzuoli F., Gori L., Lombardo G. (2007). The medicinal mushroom *Agaricus blazei* Murrill: Review of literature and pharmaco-toxicological problems. Evid.-Based Complement. Altern. Med..

[35] Hetland G., Eide D.M., Tangen J.M., Haugen M.H., Mirlashari M.R., Paulsen J.E. (2016). The *Agaricus blaze*-based mushroom extract, Andosan^TM^, protects against intestinal tumorigenesis in the A/J Min/+ mouse. PLoS ONE.

[36] Wasser S.P. (2011). Current findings, future trends, and unsolved problems in studies of medicinal mushrooms. Appl. Microbiol. Biotechnol..

[37] Macharia J.M., Zhang L., Mwangi R.W., Rozmann N., Kaposztas Z., Varjas T., Sugár M., Alfatafta H., Pintér M., Bence R.L. (2022). Are chemical compounds in medical mushrooms potent against colorectal cancer carcinogenesis and antimicrobial growth?. Cancer Cell Int..

[38] Liu J., Jia L., Kan J., Jin C.H. (2013). In vitro and in vivo antioxidant activity of ethanolic extract of white button mushroom (*Agaricus bisporus*). Food Chem. Toxicol..

[39] Mwangi R.W., Macharia J.M., Wagara I.N., Bence R.L. (2022). The antioxidant potential of different edible and medicinal mushrooms. Biomed. Pharmacother..

[40] El-Deeb N.M., Ibrahim O.M., Mohamed M.A., Farag M.M.S., Farrag A.A., El-Aassar M.R. (2022). Alginate/κ-carrageenan oral microcapsules loaded with *Agaricus bisporus* polysaccharides MH751906 for natural killer cells mediated colon cancer immunotherapy. Int. J. Biol. Macromol..

[41] Xu Y., Xu T., Huang C., Liu L., Kwame A.W., Zhu Y., Ren J. (2024). Preventive intervention with *Agaricus blazei* murill polysaccharide exerts anti-tumor immune effect on intraperitoneal metastasis colorectal cancer. Int. J. Biol. Macromol..

[42] Dong K., Wang J., Tang F., Liu Y., Gao L. (2024). A polysaccharide with a triple helix structure from *Agaricus bisporus*: Characterization and anti-colon cancer activity. Int. J. Biol. Macromol..

[43] He Y.Y., Liu S., Newburg D.S. (2021). Musarin, a novel protein with tyrosine kinase inhibitory activity from *Trametes versicolor*, inhibits colorectal cancer stem cell growth. Biomed. Pharmacother..

[44] Lavi I., Friesem D., Geresh S., Hadar Y., Schwartz B. (2006). An aqueous polysaccharide extract from the edible mushroom *Pleurotus ostreatus* induces anti-proliferative and pro-apoptotic effects on HT-29 colon cancer cells. Cancer Lett..

[45] Chaitanya N.S., Devi A., Sahu S., Alugoju P. (2021). Molecular mechanisms of action of Trehalose in cancer: A comprehensive review. Life Sci..

[46] Quero J., Paesa M., Morales C., Mendoza G., Osada J., Teixeira J.A., Ferreira-Santos P., Rodríguez-Yoldi M.J. (2024). Biological properties of *Boletus edulis* extract on Caco-2 cells: Antioxidant, anticancer, and anti-inflammatory effects. Antioxidants.

[47] Park G.S., Shin J., Hong S., Gopal J., Oh J.W. (2024). Anticarcinogenic potential of the mushroom polysaccharide lentinan on gastric and colon cancer cells: Antiproliferative, antitumorigenic, Mu-2-related death-inducing gene, MUDENG ramifications. J. Ind. Eng. Chem..

[48] Zhang M., Zhang Y., Zhang L., Tian Q. (2019). Mushroom polysaccharide lentinan for treating different types of cancers: A review of 12 years clinical studies in China. Prog. Mol. Biol. Translat. Sci..

[49] Fritz H., Kennedy D.A., Ishii M., Fergusson D., Fernandes R., Cooley K., Seely D. (2015). Polysaccharide K and *Coriolus versicolor* extracts for lung cancer: A systematic review. Integr. Cancer Ther..

[50] Lu H., Yang Y., Gad E., Wenner C.A., Chang A., Larson E.R., Dang Y., Martzen M., Standish L.J., Disis M.L. (2010). Polysaccharide Krestin is a novel TLR2 agonist that mediates inhibition of tumor growth via stimulation of CD8 T cells and NK cells. Clin. Cancer Res..

[51] Rosendahl A.H., Sun C., Wu D.Q., Andersson R. (2012). Polysaccharide-K (PSK) increases p21(WAF/Cip1) and promotes apoptosis in pancreatic cancer cells. Pancreatology.

[52] Camilleri E., Blundell R., Baral B., Karpiński T.M., Aruci E., Atrooz O.M. (2024). Unveiling the full spectrum of maitake mushrooms: A comprehensive review of their medicinal, therapeutic, nutraceutical, and cosmetic potential. Heliyon.

[53] Fontes Vieira P.A., Gontijo D.C., Vieira B.C., Fontes E.A.F., de Assunção L.S., Leite J.P.V., Oliveira M.G.D.A., Kasuya M.C.M. (2013). Antioxidant activities, total phenolics and metal contents in *Pleurotus ostreatus* mushrooms enriched with iron, zinc or lithium. LWT.

[54] Kodama N., Komuta K., Sakai N., Nanba H. (2002). Effects of D-Fraction, a polysaccharide from *Grifola frondosa* on tumor growth involve activation of NK cells. Biol. Pharm. Bull..

[55] Zhao F., Guo Z., Ma Z.R., Ma L.L., Zhao J. (2021). Antitumor activities of *Grifola frondosa* (Maitake) polysaccharide: A meta-analysis based on preclinical evidence and quality assessment. J. Ethnopharmacol..

[56] Brown G.D., Gordon S. (2001). Immune recognition. A new receptor for beta-glucans. Nature.

[57] Lemieszek M., Rzeski W. (2012). Anticancer properties of polysaccharides isolated from fungi of the Basidiomycetes class. Contemp. Oncol..

[58] Yuan B., Ma N., Zhao L., Zhao E., Gao Z., Wang W., Song M., Zhang G., Hu Q., Xiao H. (2017). In vitro and in vivo inhibitory effects of a *Pleurotus eryngii* protein on colon cancer cells. Food Funct..

[59] Yuan B., Zhao L., Rakariyatham K., Han Y., Gao Z., Muinde Kimatu B., Hu Q., Xiao H. (2017). Isolation of a novel bioactive protein from an edible mushroom *Pleurotus eryngii* and its anti-inflammatory potential. Food Funct..

[60] Chen C.H., Wu J.Y., Chen C.H., Chang W.H., Chung K.T., Liu Y.W., Lu F.J. (2011). Anti-cancer effects of protein extracts from *Calvatia lilacina, Pleurotus ostreatus* and *Volvariella volvacea*. Evid. Based Complement. Altern. Med..

[61] Rezvani V., Pourianfar H.R., Mohammadnejad S., Madjid Ansari A., Farahmand L. (2020). Anticancer potentiality and mode of action of low-carbohydrate proteins and peptides from mushrooms. Appl. Microbiol. Biotechnol..

[62] Maiti S., Bhutia S.K., Mallick S.K., Kumar A., Khadgi N., Maiti T.K. (2008). Antiproliferative and immunostimulatory protein fraction from edible mushrooms. Environ. Toxicol. Pharm..

[63] Evans R., Rhodes J., Kinsella A. (1995). Mushroom lectin inhibits invasion of a human colon cancer cell line into collagen gels. Clin. Sci..

[64] Evans R.C., Fear S., Ashby D., Williams E., Van der Vliet M., Rhodes J.M., Dunstan F.D.J. (2002). Diet and colorectal cancer: An investigation of the lectin/galactose hypothesis. Gastroenterology.

[65] Yu L.G., Fernig D.G., White M.R.H., Spiller D.G., Appleton P., Evans R.C., Grierson I., Smith J.A., Davies H., Gerasimenko O.V. (1999). Edible mushroom (*Agaricus bisporus*) lectin, which reversibly inhibits epithelial cell proliferation, blocks nuclear localization sequence-dependent nuclear protein import. J. Biol. Chem..

[66] Zhou R., Liu Z.K., Zhang Y.N., Wong J.H., Ng T.B., Liu F. (2018). Research progress of bioactive proteins from the edible and medicinal mushrooms. Curr. Prot. Peptide Sci..

[67] Singh S.S., Wang H., Chan Y.S., Pan W., Dan X., Yin C.M., Akkouh O., Ng T.B. (2014). Lectins from edible mushrooms. Molecules.

[68] Camilleri E., Blundell R., Baral B., Karpiński T.M., Aruci E., Atrooz O.M. (2024). A comprehensive review of the health benefits, phytochemicals, and enzymatic constituents for potential therapeutic and industrial applications of turkey tail mushrooms. Discov. Appl. Sci..

[69] Sun J., Chen Q.J., Zhu M.J., Wang H.X., Zhang G.Q. (2014). An extracellular laccase with antiproliferative activity from the Sanghuang mushroom *Inonotus baumii*. J. Mol. Catal. B Enzym..

[70] Yuzugullu Karakus Y., Isik S., Kale Bakir E., Turkmenoglu A., Deveci Ozkan A. (2025). Characterization of the three-phase partitioned laccase from *Trametes versicolor* strain with antiproliferative activity against breast cancer cells. Int. J. Biol. Macromol..

[71] Kim M.Y., Seguin P., Ahn J.K., Kim J.J., Chun S.C., Kim E.H., Seo S.H., Kang E.Y., Kim S.L., Park Y.J. (2008). Phenolic compound concentration and antioxidant activities of edible and medicinal mushrooms from Korea. J. Agric. Food Chem..

[72] Paloi S., Kumla J., Paloi B.P., Srinuanpan S., Hoijang S., Karunarathna S.C., Acharya K., Suwannarach N., Lumyong S. (2023). Termite mushrooms (Termitomyces), a potential source of nutrients and bioactive compounds exhibiting human health benefits: A review. J. Fungi.

[73] Silva M., Lageiro M., Ramos A.C., Reboredo F.H., Gonçalves E.M. (2024). Cultivated mushrooms: A comparative study of antioxidant activity and phenolic content. Biol. Life Sci. Forum.

[74] Chu M., Khan R.D., Zhou Y., Agar O.T., Barrow C.J., Dunshea F.R., Suleria H.A.R. (2023). LC-ESI-QTOF-MS/MS characterization of phenolic compounds in common commercial mushrooms and their potential antioxidant activities. Processes.

[75] Durgo K., Koncar M., Komes D., Belscak-Cvitanovic A., Franekic J., Jakopovich I., Jakopovich N., Jakopovich B. (2013). Cytotoxicity of blended versus single medicinal mushroom extracts on human cancer cell lines: Contribution of polyphenol and polysaccharide content. Int. J. Med. Mushrooms.

[76] Palacios I., Lozano M., Moro C., D’Arrigo M., Rostagno M.A., Martínez J.A., García-Lafuente A., Guillamón E., Villares A. (2011). Antioxidant properties of phenolic compounds occurring in edible mushrooms. Food Chem..

[77] Lin S., Ching L.T., Chen J., Cheung P.C.K. (2015). Antioxidant and anti-angiogenic effects of mushroom phenolics-rich fractions. J. Funct. Foods.

[78] Park H.J. (2022). Current uses of mushrooms in cancer treatment and their anticancer mechanisms. Int. J. Mol. Sci..

[79] Chang T.C., Yeh C.T., Adebayo B.O., Lin Y.C., Deng L., Rao Y.K., Huang C.C., Lee W.H., Wu A.T.H., Hsiao M. (2015). 4-Acetylantroquinonol B inhibits colorectal cancer tumorigenesis and suppresses cancer stem-like phenotype. Toxicol. Appl. Pharmacol..

[80] Chu Y.C., Tsai T.-Y., Yadav V.K., Deng L., Huang C.-C., Tzeng Y.-M., Yeh C.-T., Chen M.-Y. (2021). 4-Acetyl-Antroquinonol B improves the sensitization of cetuximab on both Kras mutant and wild type colorectal cancer by modulating the expression of Ras/Raf/miR-193a-3p signaling axis. Int. J. Mol. Sci..

[81] Ayeka P.A. (2018). Potential of mushroom compounds as immunomodulators in cancer immunotherapy: A review. Evid. Based Complement. Altern. Med..

[82] Dan A., Swain R., Belonce S., Jacobs R.J. (2023). Therapeutic effects of medicinal mushrooms on gastric, breast, and colorectal cancer: A scoping review. Cureus.

[83] De Silva D.D., Rapior S., Fons F., Bahkali A.H., Hyde K.D. (2012). Medicinal mushrooms in supportive cancer therapies: An approach to anti-cancer effects and putative mechanisms of action. Fungal Divers..

[84] Figueiredo L., Régis W.C.B. (2017). Medicinal mushrooms in adjuvant cancer therapies: An approach to anticancer effects and presumed mechanisms of action. Nutrire.

[85] Subramanian R.R., Yamakawa A. (2012). Combination therapy targeting Raf-1 and MEK causes apoptosis of HCT116 colon cancer cells. Int. J. Oncol..

[86] Lavi I., Nimri L., Levinson D., Peri I., Hadar Y., Schwartz B. (2012). Glucans from the edible mushroom *Pleurotus pulmonarius* inhibit colitis-associated colon carcinogenesis in mice. J. Gastroenterol..

[87] Zaila C.S., Zuraina M.F., Norfazlina M.N., Mun L.L., Nurshahirah N., Florinsiah L., Rajab N.F. (2013). Antiproliferative effect of *Lignosus rhinocerotis*, the Tiger Milk mushroom on HCT 116 human colorectal cancer cells. Open Conf. Proc. J..

[88] Aakif M., Balfe P., Elfaedy O., Awan F.N., Pretorius F., Silvio L., Castinera C., Mustafa H. (2016). Study on colorectal cancer presentation, treatment and follow-up. Int. J. Color. Dis..

[89] Li J., Huang L., Zhao H., Yan Y., Lu J. (2020). The role of interleukins in colorectal cancer. Int. J. Biol. Sci..

[90] Bergman M., Levin G.S., Bessler H., Djaldetti M., Salman H. (2013). Resveratrol affects the cross talk between immune and colon cancer cells. Biomed. Pharmacother..

[91] Pastille E., Wasmer M.H., Adamczyk A., Vu V.P., Mager L.F., Phuong N.N.T., Palmieri V., Simillion C., Hansen W., Kasper S. (2019). The IL-33/ST2 pathway shapes the regulatory T cell phenotype to promote intestinal cancer. Mucosal Immunol..

[92] Al Obeed O.A., Alkhayal K.A., Al Sheikh A., Zubaidi A.M., Vaali-Mohammed M.A., Boushey R., Mckerrow J.H., Abdulla M.H. (2014). Increased expression of tumor necrosis factor-α is associated with advanced colorectal cancer stages. World J. Gastroenterol. WJG.

[93] Zhao P., Zhang Z. (2018). TNF-α promotes colon cancer cell migration and invasion by upregulating TROP-2. Oncol. Lett..

[94] Liu X., Li Y., Sun X., Muftuoglu Y., Wang B., Yu T., Hu Y., Ma L., Xiang M., Guo G. (2018). Powerful anti-colon cancer effect of modified nanoparticle-mediated IL-15 immunogene therapy through activation of the host immune system. Theranostics.

[95] Waldmann T.A. (2006). The biology of interleukin-2 and interleukin-15: Implications for cancer therapy and vaccine design. Nat. Rev. Immunol..

[96] Sharma D., Malik A., Guy C.S., Karki R., Vogel P., Kanneganti T.D. (2017). Pyrin inflammasome regulates tight junction integrity to restrict colitis and tumorigenesis. Gastroenterology.

[97] Muszyńska B., Grzywacz A., Kała K., Gdula-Argasińska J. (2018). Anti-inflammatory potential of in vitro cultures of the white button mushroom, *Agaricus bisporus* (Agaricomycetes), in Caco-2 Cells. Int. J. Med. Mushrooms.

[98] Chaudhary P., Janmeda P., Docea A.O., Yeskaliyeva B., Abdull Razis A.F., Modu B., Calina D., Sharifi-Rad J. (2023). Oxidative stress, free radicals and antioxidants: Potential crosstalk in the pathophysiology of human diseases. Front. Chem..

[99] Muchtaridi M., Az-Zahra F., Wongso H., Setyawati L.U., Novitasari D., Ikram E.H.K. (2024). Molecular mechanism of natural food antioxidants to regulate ROS in treating cancer: A review. Antioxidants.

[100] Pham-Huy L.A., He H., Pham-Huy C. (2008). Free radicals, antioxidants in disease and health. Int. J. Biomed. Sci..

[101] Baeuerle P.A., Henkel T. (1994). Function and activation of NF-KappaB in the immune system. Annu. Rev. Immunol..

[102] Barkett M., Gilmore T.D. (1999). Control of apoptosis by Rel/NF-κB transcription factors. Oncogene.

[103] Dolcet X., Llobet D., Pallares J., Matias-Guiu X. (2005). NF-kB in development and progression of human cancer. Virchows Arch..

[104] Wan F., Lenardo M.J. (2009). Specification of DNA binding activity of NF- B proteins. Cold Spring Harb. Perspect. Biol..

[105] Chen Y., Chen M., Deng K. (2022). Blocking the Wnt/β-catenin signaling pathway to treat colorectal cancer: Strategies to improve current therapies (Review). Int. J. Oncol..

[106] He K., Gan W.J. (2023). Wnt/β-Catenin signaling pathway in the development and progression of colorectal cancer. Cancer Manag. Res..

[107] Heslin M.J., Yan J., Johnson M.R., Weiss H., Diasio R.B., Urist M.M. (2001). Role of matrix metalloproteinases in colorectal carcinogenesis. Ann. Surg..

[108] Said A.H., Raufman J.P., Xie G. (2014). The role of matrix metalloproteinases in colorectal cancer. Cancers.

[109] Pfeffer C.M., Singh A.T.K. (2018). Apoptosis: A target for anticancer therapy. Int. J. Mol. Sci..

[110] Xu J., Shen R., Jiao Z., Chen W., Peng D., Wang L., Yu N., Peng C., Cai B., Song H. (2022). Current advancements in antitumor properties and mechanisms of medicinal components in edible mushrooms. Nutrients.

[111] Ramesh P., Medema J.P. (2020). BCL-2 family deregulation in colorectal cancer: Potential for BH3 mimetics in therapy. Apoptosis.

[112] Güllülü Ö., Hehlgans S., Rödel C., Fokas E., Rödel F. (2021). Tumor suppressor protein p53 and inhibitor of apoptosis proteins in colorectal cancer—A promising signaling network for therapeutic interventions. Cancers.

[113] Zhou M., Liu X., Li Z., Huang Q., Li F., Li C.Y. (2018). Caspase-3 regulates the migration, invasion, and metastasis of colon cancer cells. Int. J. Cancer.

[114] Baek S.J., Kim J.S., Jackson F.R., Eling T.E., McEntee M.F., Lee S.H. (2004). Epicatechin gallate-induced expression of NAG-1 is associated with growth inhibition and apoptosis in colon cancer cells. Carcinogenesis.

[115] Kim I.Y., Park S.Y., Kang Y., Thapa D., Choi H.G., Kim J.A. (2011). Role of nonsteroidal anti-inflammatory drug-activated gene-1 in docetaxel-induced cell death of human colorectal cancer cells with different p53 status. Arch. Pharm. Res..

[116] Krieg A., Werner T.A., Verde P.E., Stoecklein N.H., Knoefel W.T. (2013). Prognostic and clinicopathological significance of survivin in colorectal cancer: A meta-analysis. PLoS ONE.

[117] Li G., Wang Y., Wang W., Lv G., Li X., Wang J., Liu X., Yuan D., Deng S., You D. (2024). BIRC5 as a prognostic and diagnostic biomarker in pan-cancer: An integrated analysis of expression, immune subtypes, and functional networks. Front. Genet..

[118] Gariboldi M.B., Marras E., Ferrario N., Vivona V., Prini P., Vignati F., Perletti G. (2023). Anti-cancer potential of edible/medicinal mushrooms in breast cancer. Int. J. Mol. Sci..

[119] Jakopovic B., Oršolić N., Jakopovich I. (2021). Proteomic research on the antitumor properties of medicinal mushrooms. Molecules.

[120] Randeni N., Xu B. (2024). New insights into signaling pathways of cancer prevention effects of polysaccharides from edible and medicinal mushrooms. Phytomedicine.

[121] Jakopovic B., Horvatić A., Klobučar M., Gelemanović A., Grbčić P., Oršolić N., Jakopovich I., Pavelić S.K. (2020). Treatment with medicinal mushroom extract mixture inhibits translation and reprograms metabolism in advanced colorectal cancer animal model as evidenced by tandem mass Tags proteomics analysis. Front. Pharmacol..

[122] Jiang Y.A., Luo H.S., Zhang Y.Y., Fan L.F., Jiang C.Q., Chen W.J. (2003). Telomerase activity and cell apoptosis in colon cancer cell by human telomerase reverse transcriptase gene antisense oligodeoxynucleotide. World J. Gastroenterol..

[123] Simsek B.C., Pehlivan S., Karaoglu A. (2010). Human telomerase reverse transcriptase expression in colorectal tumors: Correlations with immunohistochemical expression and clinicopathologic features. Ann. Diagn. Pathol..

[124] Huo J., Nie K., Yang T., Zhang S., Zhu Z., Peng X., Zhang Y. (2025). Network pharmacology combined with transcriptomics reveals that *Ganoderma lucidum* spore and *Sanghuangporus vaninii* compound extract exerts anti-colorectal cancer effects via CYP24A1-mediated VDR pathway and TERT-mediated Wnt signaling pathway. J. Ethnopharmacol..

[125] Liao C., Hsiao Y., Hsu C., Lin M., Wang J.C., Huang Y., Ko J. (2006). Transcriptionally mediated inhibition of telomerase of fungal immunomodulatory protein from *Ganoderma tsugae* in A549 human lung adenocarcinoma cell line. Mol. Carcinog..

[126] Dang D.T., Chen F., Gardner L.B., Cummins J.M., Rago C., Bunz F., Kantsevoy S.V., Dang L.H. (2006). Hypoxia-inducible factor-1alpha promotes nonhypoxia-mediated proliferation in colon cancer cells and xenografts. Cancer Res..

[127] Ioannou M., Paraskeva E., Baxevanidou K., Simos G., Papamichali R., Papacharalambous C., Samara M., Koukoulis G. (2015). HIF-1α in colorectal carcinoma: Review of the literature. J. Buon..

[128] Kirdeeva Y., Fedorova O., Daks A., Barlev N., Shuvalov O. (2022). How should the worldwide knowledge of traditional cancer healing be integrated with herbs and mushrooms into modern molecular pharmacology?. Pharmaceuticals.

[129] Maithani R., Tulsawani R. (2025). Lingzhi or Reishi medicinal mushroom (agaricomycetes) *Ganoderma lucidum* aqueous extract reverses hypoxia induced redox imbalance and inflammatory response in rat thymocytes. Int. J. Med. Mushrooms.

[130] Cao D., Qin S., Mu Y., Zhong M. (2017). The role of MRP1 in the multidrug resistance of colorectal cancer. Oncol. Lett..

[131] Jiang Z., Jin T., Gao F., Liu J., Zhong J., Zhao H. (2011). Effects of ganoderic acid Me on inhibiting multidrug resistance and inducing apoptosis in multidrug resistant colon cancer cells. Process Biochem..

[132] Chang S.T., Wasser S.P. (2018). Current and future research trends in agricultural and biomedical applications of medicinal mushrooms and mushroom products (Review). Int. J. Med. Mushrooms.

[133] Wasser S.P. (2010). Medicinal mushroom science: History, current status, future trends, and unsolved problems. Int. J. Med. Mushrooms.

[134] Jeitler M., Michalsen A., Frings D., Hübner M., Fischer M., Koppold-Liebscher D.A., Murthy V., Kessler C.S. (2020). Significance of medicinal mushrooms in integrative oncology: A narrative review. Fron. Pharmacol..

[135] Islam M.R., Akash S., Rahman M.M., Nowrin F.T., Akter T., Shohag S., Rauf A., Aljohani A.S.M., Simal-Gandara J. (2022). Colon cancer and colorectal cancer: Prevention and treatment by potential natural products. Chem. Biol. Inter..

[136] Patel S., Goyal A. (2012). Recent developments in mushrooms as anti-cancer therapeutics: A review. 3 Biotech.

[137] Paul D., Sanap G., Shenoy S., Kalyane D., Kalia K., Tekade R.K. (2021). Artificial intelligence in drug discovery and development. Drug Discov. Today.

[138] Leal K., Rojas E., Madariaga D., Contreras M.J., Nuñez-Montero K., Barrientos L., Goméz-Espinoza O., Iturrieta-González I. (2024). Unlocking fungal potential: The CRISPR-Cas system as a strategy for secondary metabolite discovery. J. Fungi.

[139] Kwong K.W., Xin Y., Lai N.C., Sung J.C., Wu K.C., Hamied Y.K., Sze E.T., Lam D.M. (2023). Oral Vaccines: A Better Future of Immunization. Vaccines.

[140] Pérez-Martínez A.S., Acevedo-Padilla S.A., Bibbins-Martínez M., Galván-Alonso J., Rosales-Mendoza S. (2015). A perspective on the use of *Pleurotus* for the development of convenient fungi-made oral subunit vaccines. Vaccine.

[141] Uddin M.S., Kaldis A., Menassa R., Ortiz Guluarte J., Barreda D.R., Guan L.L., Alexander T.W. (2024). Mucosal immunization with spore-based vaccines against *Mannheimia haemolytica* enhances antigen-specific immunity. Vaccines.

